# Supplementary material for: A humanized NOVA1 splicing factor alters mouse vocal communications
Source: Nat Commun. 2025 Feb 18;16:1542. doi: 10.1038/s41467-025-56579-2 (PMC11836289; doi:10.1038/s41467-025-56579-2)
Supplement: Supplementary file 1 — Supplementary Information [file 41467_2025_56579_MOESM1_ESM.pdf]

Supplementary information for

A humanized NOVA1 splicing factor alters mouse vocal communications

Yoko Tajima*, César D. M. Vargas, Keiichi Ito, Wei Wang, Ji-Dung Luo, Jiawei Xing, Nurdan Kuru, Luiz Carlos Machado, Adam Siepel, Thomas S. Carroll, Erich D. Jarvis, Robert B. Darnell*

*Corresponding author. Yoko Tajima: ytajima@rockefeller.edu ; Robert B. Darnell: darnelr@rockefeller.edu

The PDF file includes:

Supplementary Figure 1 to 17
Supplementary References

Other Supplementary Materials for this manuscript include the following:

Supplementary Data 1 - 18

a

rs762662114

Organism	Homo sapiens
Position	chr14:26448894 (GRCh38.p14)
Gene	NOVA1
Consequence	Missense Variant
Alleles	C>T
Type	SNV Single Nucleotide Variation

Allele Frequency (alternate allele / total allele number, project name):

T=0.000004	(1/264690, TOPMED)
T=0.000012	(3/250246, GnomAD_exome)
T=0.000017	(2/120984, ExAC)
T=0.00000	(0/14050, ALFA)
T=0.00	(0/88, Ancient Sardinia)

[dbSNP, Build 156, Released September 21, 2022]

b

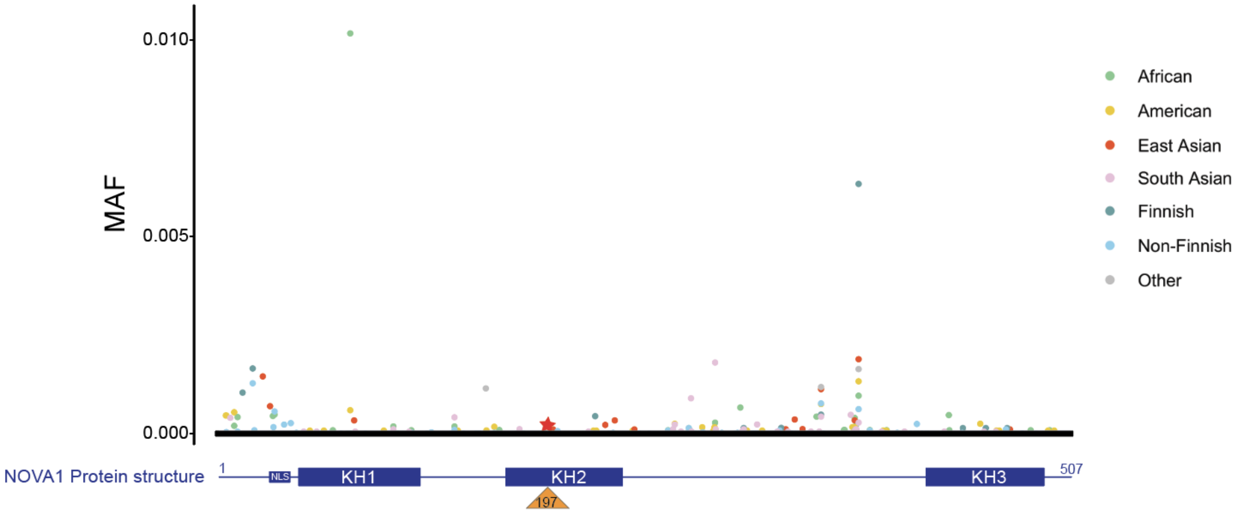

Supplementary Fig. 1

(**a**) The SNP report from dbSNP database. The single nucleotide variation (rs762662114) responsible for modern human specific amino acid in NOVA1 (197th Val). Alternate allele frequencies in the total number of samples for each genome project are shown. (**b**) MAF analysis in the NOVA1 gene across human ancestries. SNPs detected in the genome analysis of 121,410 modern humans on the NOVA1 CDS, along with the NOVA1 protein structure. Each SNP is color-coded by ethnicity. SNP corresponding to the 197th amino acid (ancient human-type variant) is indicated by a star. Source data are provided as a Source Data file.

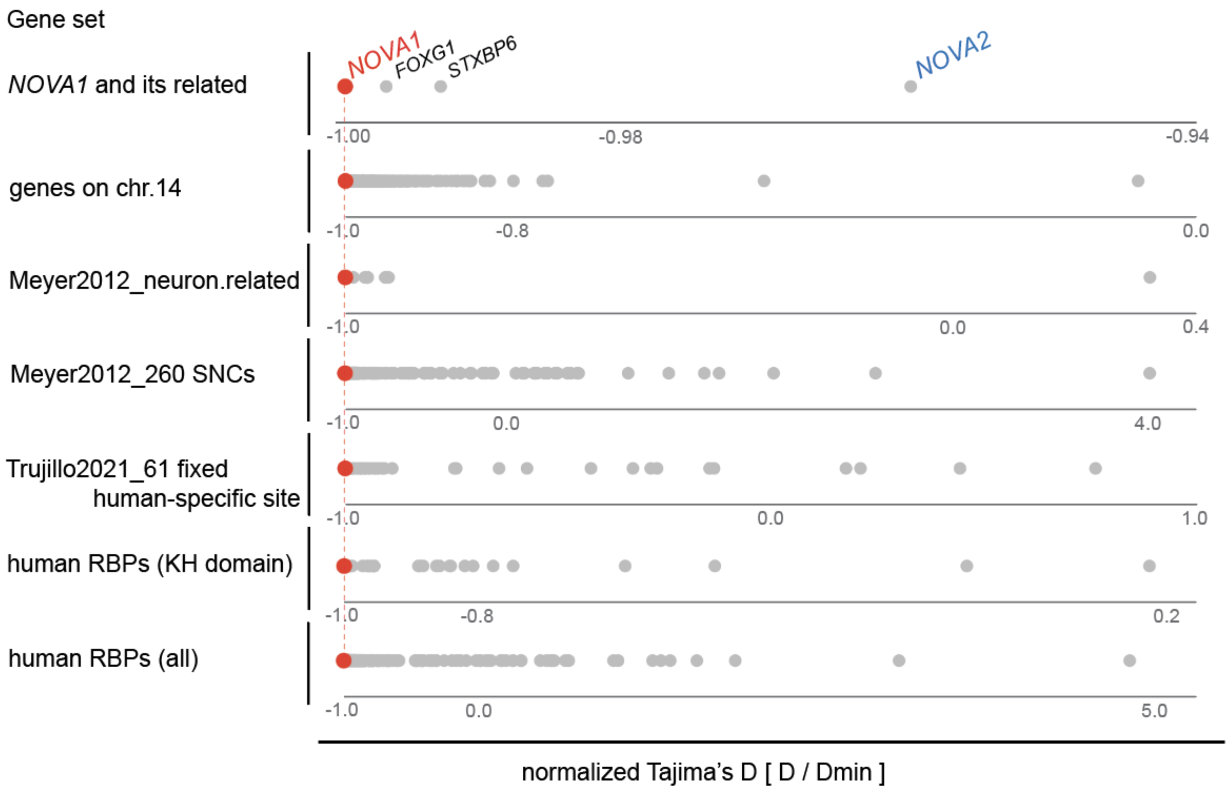

Supplementary Fig. 2

Comparison of Normalized Tajima's D values. The closer the normalized Tajima's D value approaches -1, the greater the likelihood that the gene has undergone strong purifying selection[1]. The first gene set includes *NOVA1* and *NOVA2*, and closest protein-coding *NOVA1*-neighboring genes (*FOXG1* and *STXBP6*) on chr14. Each gray dot indicates a gene in each gene list. The normalized Tajima's D value for *NOVA1* (red) is -0.9993. The second gene set includes all genes on chr.14 where *NOVA1* is located. These gene sets are also shown in Figure 1d. The third and fourth gene sets are based on Meyer's report[2], with genes related to the 260 human-specific single-nucleotide changes (SNCs) that cause fixed amino acid substitutions in well-defined human coding sequence, or to the subset of these genes (eight among the 260 SNCs) whose function is associated with brain function or nervous system development. The fifth gene set is based on Trujillo's report[3], with genes associated with 61 autosomal fixed derived mutations in all humans compared to Neanderthal genomes and Denisovan genome. The sixth and seventh gene sets include human RNA binding proteins (RBPs) with KH domain or all annotated RBPs.

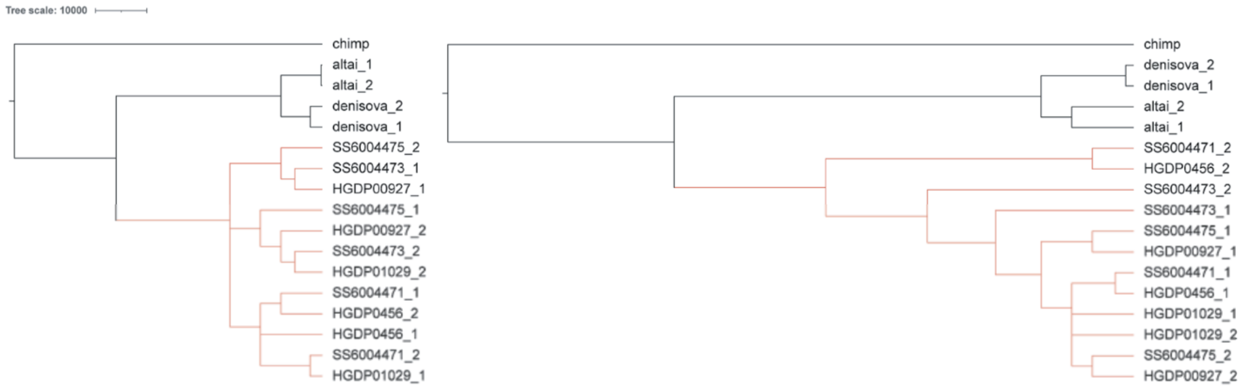

Supplementary Fig. 3

Recent burst of coalescence of a sweep for NOVA1 197V. The left tree shows the *NOVA1* SNP, with the shortened branches and burst of relatively recent coalescence events leading to the modern humans (reflecting a rise in frequency of the derived allele). The right tree shows *FRMD8* for contrast, with longer branches and delayed coalescence: more typical of what would be expected in the absence of a selective sweep. The sampled ARGs included two Yoruba (HGDP00927, SS6004475), two Mbuti (SS6004471, HGDP0456), and two San (HGDP01029, SS6004473) individuals, as well as the Altai Neanderthal and Denisovan sequences and a chimpanzee outgroup (panTro4). The numbers following the samples represent the separate haplotypes from each individual. Red lines indicate derived allele.

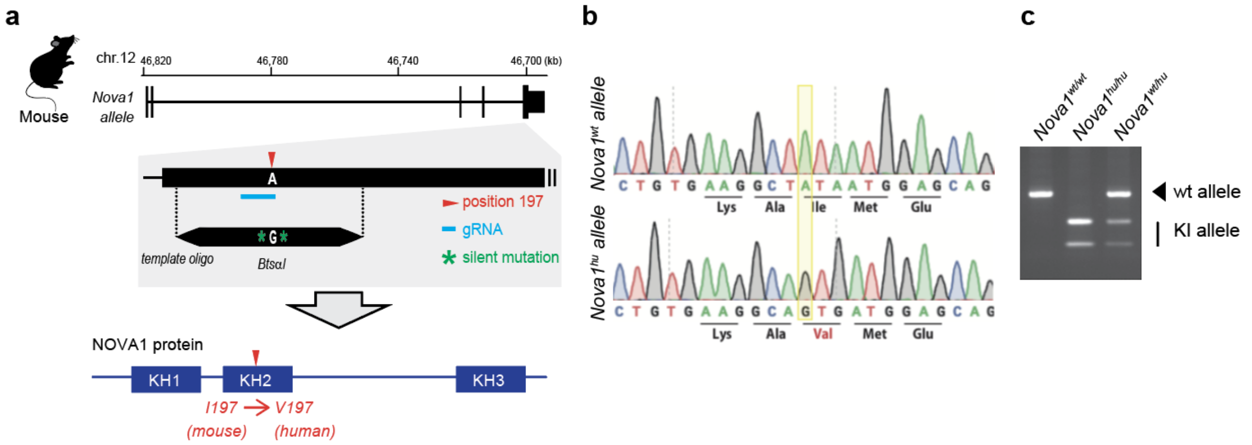

d

gRNA for generation of humanized NOVA1 knock-in mice
[CRISPRdirect]
Genome
Mouse (Mus musculus) genome, GRCm39/mm39 (Jun, 2020)

gRNA sequence	pam sequence	hit_20mer	hit_12mer
TGCTACTGTGAAGGCTATAATGG	NGG	1	11

Target Site: 20mer+PAM

chr.	strand	start	end	sbjct	match	Name	Gene	region
chr12	-	46747681	46747703	CCATTATAGCCTTCA	23	Target Site	NOVA1	CDS

Potential Off Target Sites: 12mer+PAM

chr.	strand	start	end	sbjct	match	Name	Gene	region
chr2	+	141303564	141303578	TGAAGGCTATAACGG	15	OT1	Macrod2	intron
chr5	+	71203878	71203892	TGAAGGCTATAATGG	15	OT2	Gabra2	intron
chr6	+	14018751	14018765	TGAAGGCTATAAAGG	15	OT3	AK143417	intron
chr7	+	127986246	127986260	TGAAGGCTATAAAGG	15	OT4	Rgs10	intron
chr8	+	6037054	6037068	TGAAGGCTATAAAGG	15	OT5	upstream of Gm44843	deep intergenic
chr9	+	34818427	34818441	TGAAGGCTATAAGGG	15	OT6	Kirrel3	intron
chr17	+	7424358	7424372	TGAAGGCTATAAGGG	15	OT7	Rps6ka2	intron
chr1	-	58770592	58770606	CCTTTATAGCCTTCA	15	OT8	Cflar	intron
chr3	-	143183496	143183510	CCTTTATAGCCTTCA	15	OT9	downstream of 1700001N15Rik	deep intergenic
chr12	-	46747681	46747695	CCATTATAGCCTTCA	15	Target Site	NOVA1	CDS
chr18	-	40219960	40219974	CCTTTATAGCCTTCA	15	OT11	Gm50395	intron

Supplementary Fig. 4

Generation of humanized *Nova1* mice (*Nova1*$^{hu/hu}$). (**a**) Overview of the strategy to generate mice with a modern human-specific amino acid substitution in NOVA1 protein. Using CRISPR/Cas9, nucleotide substitutions (that lead to a single amino acid change from Isoleucine to Valine) are introduced. Two silent mutations were introduced for genotyping. (**b**) DNA sequencing of *Nova1* allele in wild-type mice and in mice in which correct knock-in was introduced (*Nova1*^{hu}). (**c**) Genotyping of *Nova1*$^{hu/hu}$ mice using restriction enzymes. The introduction of silent mutations creates a *BtsIα* recognition site. *Nova1*^{hu} allele is distinguished from wild-type *Nova1* allele by restriction enzyme treatment after PCR. (**d**) The gRNA sequence and predicted off-target site information predicted by CRISPR direct (https://crispr.dbcls.jp/). There are 10 potential off target loci with mismatches outside of the PAM+12mer core sequences. (**e**) The genomic sequencing of the potential off target (POT) loci. Alignment of each genotype and reference genome for the genomic sequence of 100 bases around the POTs are shown. Asterisks indicate identical nucleotides. All POT sites were identical between genotypes and the reference genome, with the target site (responsible for I197V substitution) being the only detectable edits.

e

Potential Off Target (POT) Genomic Sequences

10 locations: POT1 - 10 (+ target NOVA1 site)

Subject +/- 50bp (115bp)

Alignment

- ref: reference genome (Mouse GRCm39/mm39)
- wt: NOVA1$^{wt/wt}$ mouse
- hu: NOVA1$^{hu/hu}$ mouse

∗ identical

subject (12mer+PAM)

target site (20mer+PAM)

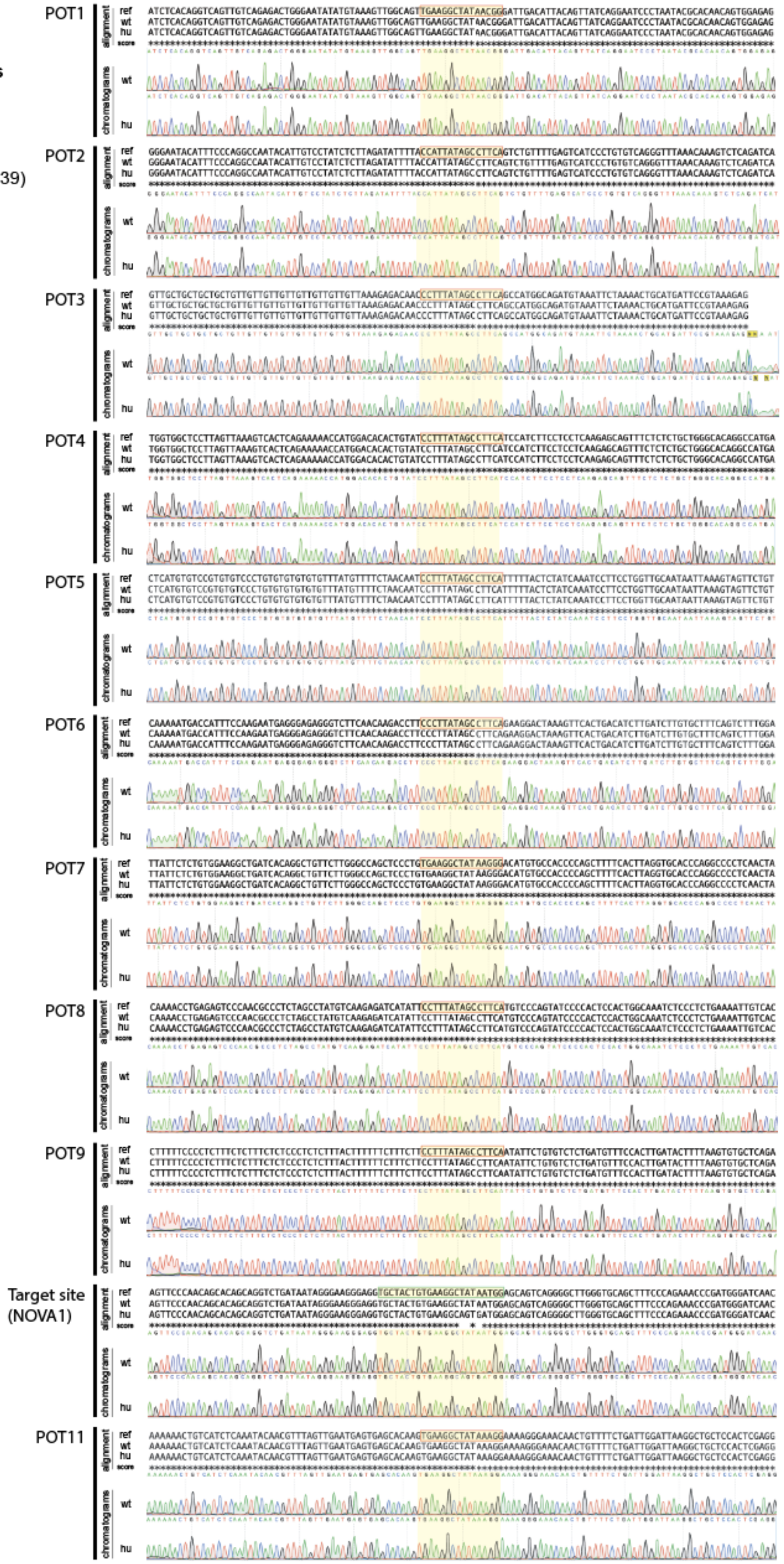

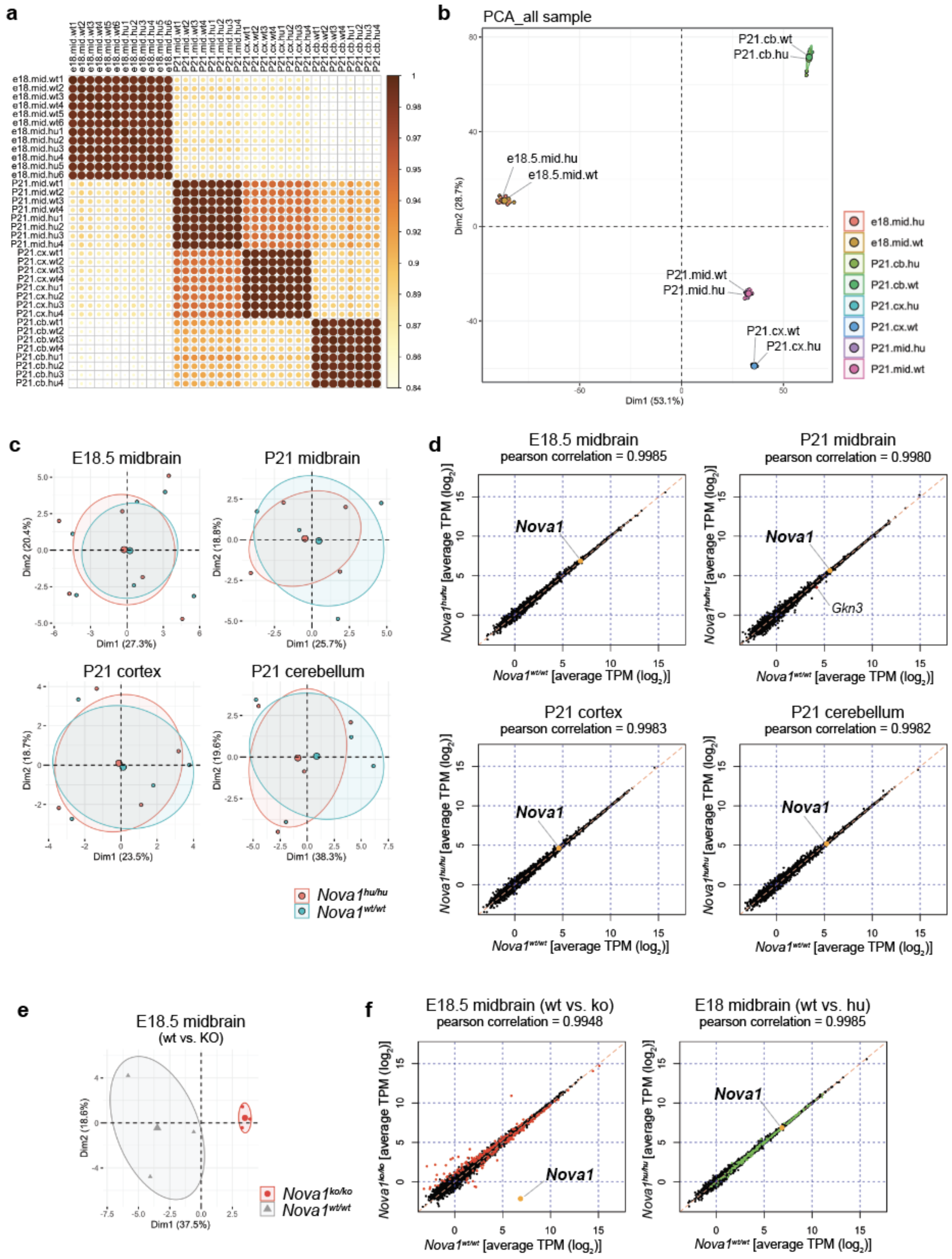

a
b
PCA_all sample
P21.cb.wt
P21.cb.hu
e18.5.mid.hu
e18.5.mid.wt
P21.mid.wt
P21.mid.hu
P21.cx.wt
P21.cx.hu
Dim1 (53.1%)
Dim2 (28.7%)
e18.mid.hu
e18.mid.wt
P21.cb.hu
P21.cb.wt
P21.cx.hu
P21.cx.wt
P21.mid.hu
P21.mid.wt
c
E18.5 midbrain
P21 midbrain
P21 cortex
P21 cerebellum
Dim1 (27.3%)
Dim2 (20.4%)
Dim1 (25.7%)
Dim2 (18.8%)
Dim1 (23.5%)
Dim2 (18.7%)
Dim1 (38.3%)
Dim2 (19.6%)
Nova1hu/hu
Nova1wt/wt
d
E18.5 midbrain
pearson correlation = 0.9985
P21 midbrain
pearson correlation = 0.9980
P21 cortex
pearson correlation = 0.9983
P21 cerebellum
pearson correlation = 0.9982
Nova1
Gkn3
Nova1wt/wt [average TPM (log2)]
Nova1hu/hu [average TPM (log2)]
e
E18.5 midbrain
(wt vs. KO)
Dim1 (37.5%)
Dim2 (18.6%)
Nova1ko/ko
Nova1wt/wt
f
E18.5 midbrain (wt vs. ko)
pearson correlation = 0.9948
E18 midbrain (wt vs. hu)
pearson correlation = 0.9985
Nova1
Nova1wt/wt [average TPM (log2)]
Nova1ko/ko [average TPM (log2)]
Nova1hu/hu [average TPM (log2)]

Supplementary Fig. 5

Comprehensive gene expression analysis in the brain. (**a**) Global correlation matrix of gene expression levels between brain samples: midbrain at E18.5, cortex, midbrain, cerebellum at P21 in *Nova1*$^{hu/hu}$ and *Nova1*$^{wt/wt}$ mice. Heatmap showing correlation coefficients for $\log_2$ (TPM+1), color intensity and the size of the circle are proportional to the correlation coefficients. (**b**) Principal component analysis of gene expression levels between samples. The X axis is the first principal component, and the Y axis is the second principal component, with a percentage of variances explained by each component approximately 53% and 29%, respectively. The ellipses indicate confidence ellipses around group mean points (large dot). (**c**) Principal component analysis of gene expression levels in each corresponding sample (age and brain region). (**d**) Gene expression correlations between *Nova1*$^{wt/wt}$ and *Nova1*$^{hu/hu}$ in corresponding brain regions and age. Scatterplots of gene expressions measured in average TPMs are shown. The axes are shown in $\log_2$ scales. The red dot indicates a differentially expressed gene between genotypes ($p<0.05$, FDR<0.1). The yellow dot indicates *Nova1* gene. Pearson correlation is reported on top of the plots. The upper two plots (midbrain) are identical with Figure 2c. (**e**) Principal component analysis of gene expression levels between *Nova1*$^{ko/ko}$ and *Nova1*$^{wt/wt}$ midbrain at E18.5. The RNA sequencing data are from GEO (GSE69711). The first principal component is 37.5%, and the second principal component is 18.6%, respectively. The ellipses indicate confidence ellipses around group mean points (large markers). (**f**) Gene expression correlations. Scatterplots of gene expressions measured in average TPMs are shown. The axes are shown in $\log_2$ scales. The yellow dot indicates *Nova1* gene. Pearson correlation is reported on top of the plots. (left) *Nova1*$^{wt/wt}$ and *Nova1*$^{ko/ko}$ at E18.5 midbrain. The red dots indicate differentially expressed genes between genotypes (FDR<0.05) (see Supplemental Table 18). (right) *Nova1*$^{wt/wt}$ and *Nova1*$^{hu/hu}$ at E18.5 midbrain (the same with top left panel in **d**). The green dots indicate differentially expressed genes in the comparison between *Nova1*$^{wt/wt}$ and *Nova1*$^{ko/ko}$ (left panel, corresponding to the red dot). The midbrain sample of E18.5, *Nova1*$^{ko/ko}$ N=3, *Nova1*$^{wt/wt}$ N=3, *Nova1*$^{hu/hu}$ N=6, *Nova1*$^{wt/wt}$ N=6. The cortex, midbrain, and cerebellum samples of P21, *Nova1*$^{hu/hu}$ N=4, *Nova1*$^{wt/wt}$ N=4, respectively.

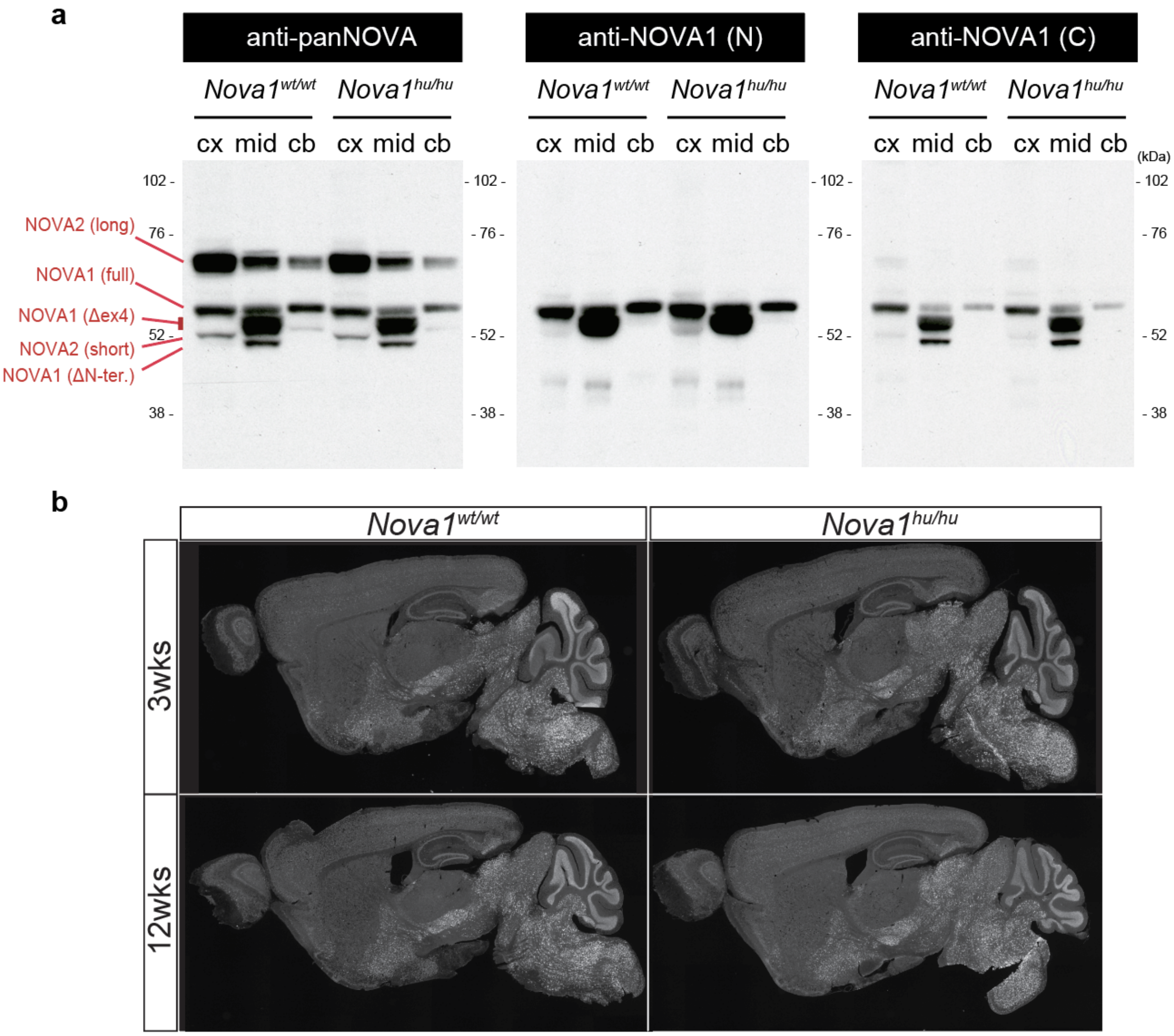

Supplementary Fig. 6

Comparison of NOVA1 protein expression between the brains of *Nova1*$^{wt/wt}$ and *Nova1*$^{hu/hu}$ mice. (**a**) Comparison of NOVA1 protein expression in dissected 3-week-old mouse brain tissues. Expression of NOVA proteins were analyzed by immunoblotted on cortex (cx), midbrain (mid), and cerebellum (cb) by panNOVA antibody, NOVA1 antibody recognizing the N-terminus and the C-terminus, respectively. The predicted NOVA1/2 protein isoforms are listed in the notes. Source data are provided as a Source Data file. (**b**) NOVA1 protein expression in *Nova1*$^{wt/wt}$ and *Nova1*$^{hu/hu}$ mice. Immunostaining for NOVA1 protein in sagittal sections of the brain at 3 and 12 weeks of age, respectively.

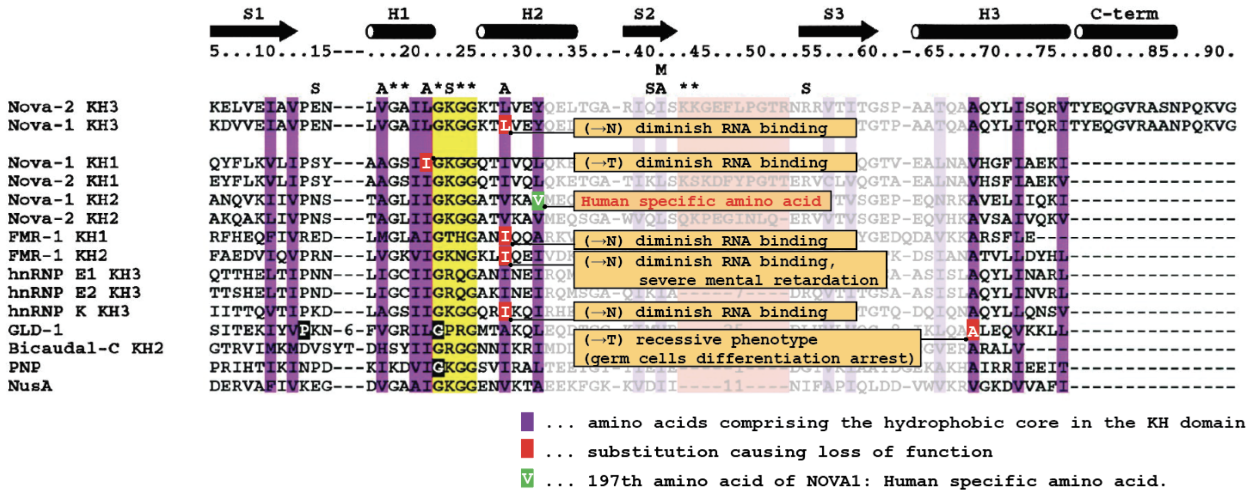

Supplementary Fig. 7

KH domain sequence alignment. Figure adapted from Lewis et al., 2000 with modifications[4]. Each RNA binding protein and comprising KH domain number are listed on the left. Secondary structural elements were based on the X-ray structure. Color coding scheme: yellow, invariant GXXG motif; purple, hydrophobic core (aliphatic α/β platform). Functional classifications: A, aliphatic stacking interaction; S, side chain–base hydrogen bond, including water-mediated contacts; M, protein backbone–base hydrogen bond; *, van der Waals contact. Amino acids on the red background are those for which loss of protein function was reported due to substitution in the hydrophobic core. Amino acids on the green background indicate the 197th valine of NOVA1, which is unique to modern humans.

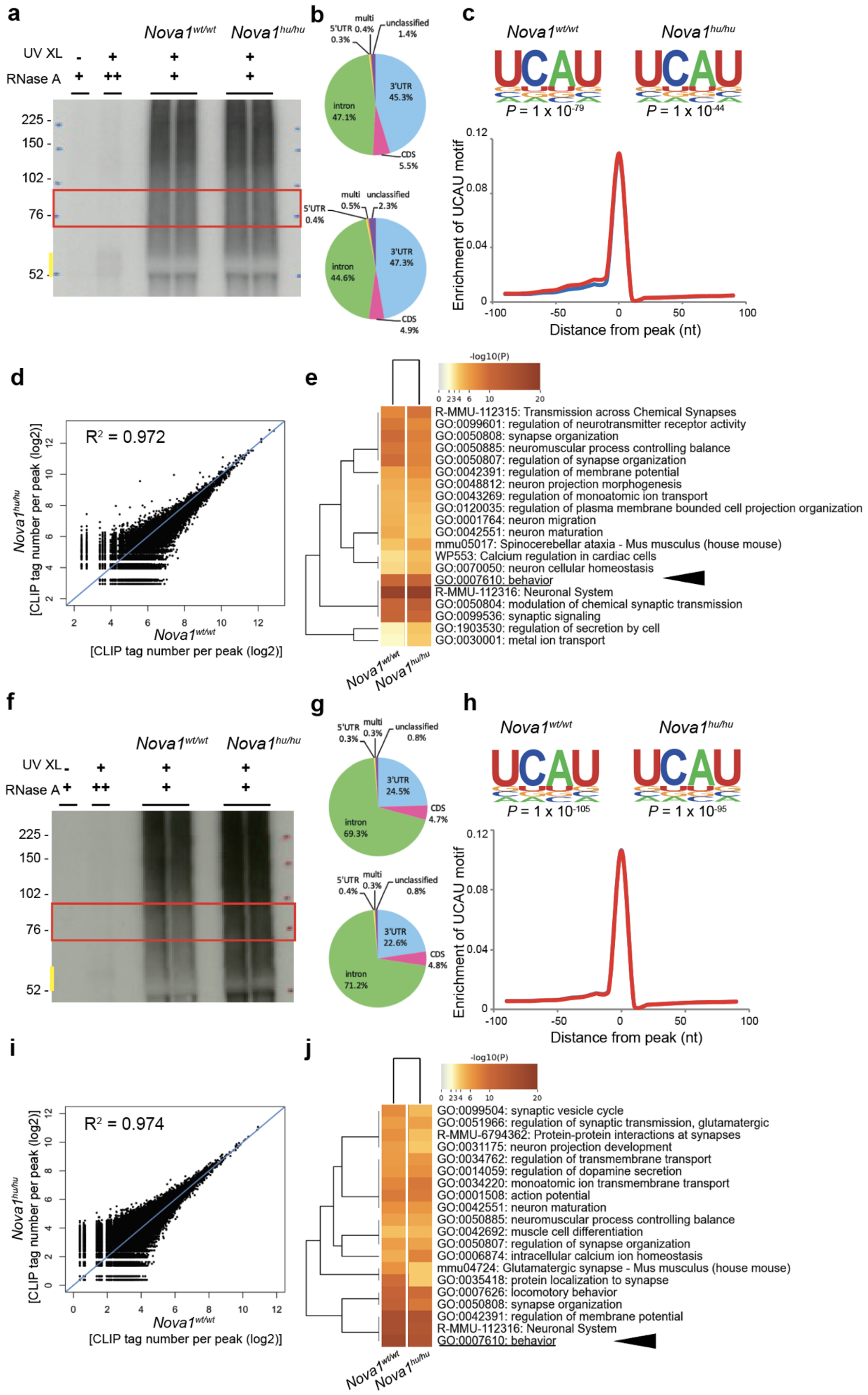
a
Nova1 wt/wt
Nova1 hu/hu
UV XL
RNase A
225
150
102
76
52
b
multi 0.4%
5'UTR 0.3%
unclassified 1.4%
3'UTR 45.3%
intron 47.1%
CDS 5.5%
multi 0.5%
unclassified 2.3%
5'UTR 0.4%
3'UTR 47.3%
intron 44.6%
CDS 4.9%
c
UCAU
P = 1 x 10-79
P = 1 x 10-44
Enrichment of UCAU motif
Distance from peak (nt)
d
R2 = 0.972
Nova1 hu/hu [CLIP tag number per peak (log2)]
Nova1 wt/wt [CLIP tag number per peak (log2)]
e
-log10(P)
R-MMU-112315: Transmission across Chemical Synapses
GO:0099601: regulation of neurotransmitter receptor activity
GO:0050808: synapse organization
GO:0050885: neuromuscular process controlling balance
GO:0050807: regulation of synapse organization
GO:0042391: regulation of membrane potential
GO:0048812: neuron projection morphogenesis
GO:0043269: regulation of monoatomic ion transport
GO:0120035: regulation of plasma membrane bounded cell projection organization
GO:0001764: neuron migration
GO:0042551: neuron maturation
mmu05017: Spinocerebellar ataxia - Mus musculus (house mouse)
WP553: Calcium regulation in cardiac cells
GO:0070050: neuron cellular homeostasis
GO:0007610: behavior
R-MMU-112316: Neuronal System
GO:0050804: modulation of chemical synaptic transmission
GO:0099536: synaptic signaling
GO:1903530: regulation of secretion by cell
GO:0030001: metal ion transport
f
g
5'UTR 0.3%
multi 0.3%
unclassified 0.8%
3'UTR 24.5%
CDS 4.7%
intron 69.3%
5'UTR 0.4%
multi 0.3%
unclassified 0.8%
3'UTR 22.6%
CDS 4.8%
intron 71.2%
h
P = 1 x 10-105
P = 1 x 10-95
i
R2 = 0.974
j
GO:0099504: synaptic vesicle cycle
GO:0051966: regulation of synaptic transmission, glutamatergic
R-MMU-6794362: Protein-protein interactions at synapses
GO:0031175: neuron projection development
GO:0034762: regulation of transmembrane transport
GO:0014059: regulation of dopamine secretion
GO:0034220: monoatomic ion transmembrane transport
GO:0001508: action potential
GO:0042551: neuron maturation
GO:0050885: neuromuscular process controlling balance
GO:0042692: muscle cell differentiation
GO:0050807: regulation of synapse organization
GO:0006874: intracellular calcium ion homeostasis
mmu04724: Glutamatergic synapse - Mus musculus (house mouse)
GO:0035418: protein localization to synapse
GO:0007626: locomotory behavior
GO:0050808: synapse organization
GO:0042391: regulation of membrane potential
R-MMU-112316: Neuronal System
GO:0007610: behavior

Supplementary Fig. 8

NOVA1-CLIP analysis in 3-week-old mouse cortex and cerebellum. (**a**-**e**) cortex samples. (**f**-**j**) cerebellum samples. (**a, f**) Representative images of autoradiography in NOVA1-CLIP of 3-week-old *Nova1*$^{wt/wt}$ and *Nova1*$^{hu/hu}$ mice. The yellow line indicates NOVA1 protein size, and the red enclosing line indicates where RNA was extracted by sectioning. Source data are provided as a Source Data file. (**b, g**) Distribution of NOVA1 CLIP peaks on the genome. (**c, h**) The most enriched binding sequence from NOVA1 CLIP peak (upper part) and frequency of that sequence (UCAU) present around the binding site (lower part). (**d, i**) Scatterplot of CLIP tag number per peak between *Nova1*$^{wt/wt}$ and *Nova1*$^{hu/hu}$. The axes are shown in $\log_2$ scales. R square value is shown. (**e**) Gene annotation analysis of NOVA1 bound transcripts. Transcripts with the top 100 peak height (read count) in each genotype were analyzed. Genes expressed in the P21 cortex were used as background for analysis. The term "behavior" is indicated with a black arrowhead. (**j**) Gene annotation analysis of NOVA1 bound transcripts. Transcripts with the top 1% peak height (read count) in each genotype were analyzed. Genes expressed in the P21 cerebellum were used as background for analysis. The term "behavior" is indicated with a black arrowhead. The cortex and cerebellum samples of P21, *Nova1*$^{hu/hu}$ N=3, *Nova1*$^{wt/wt}$ N=3, respectively.

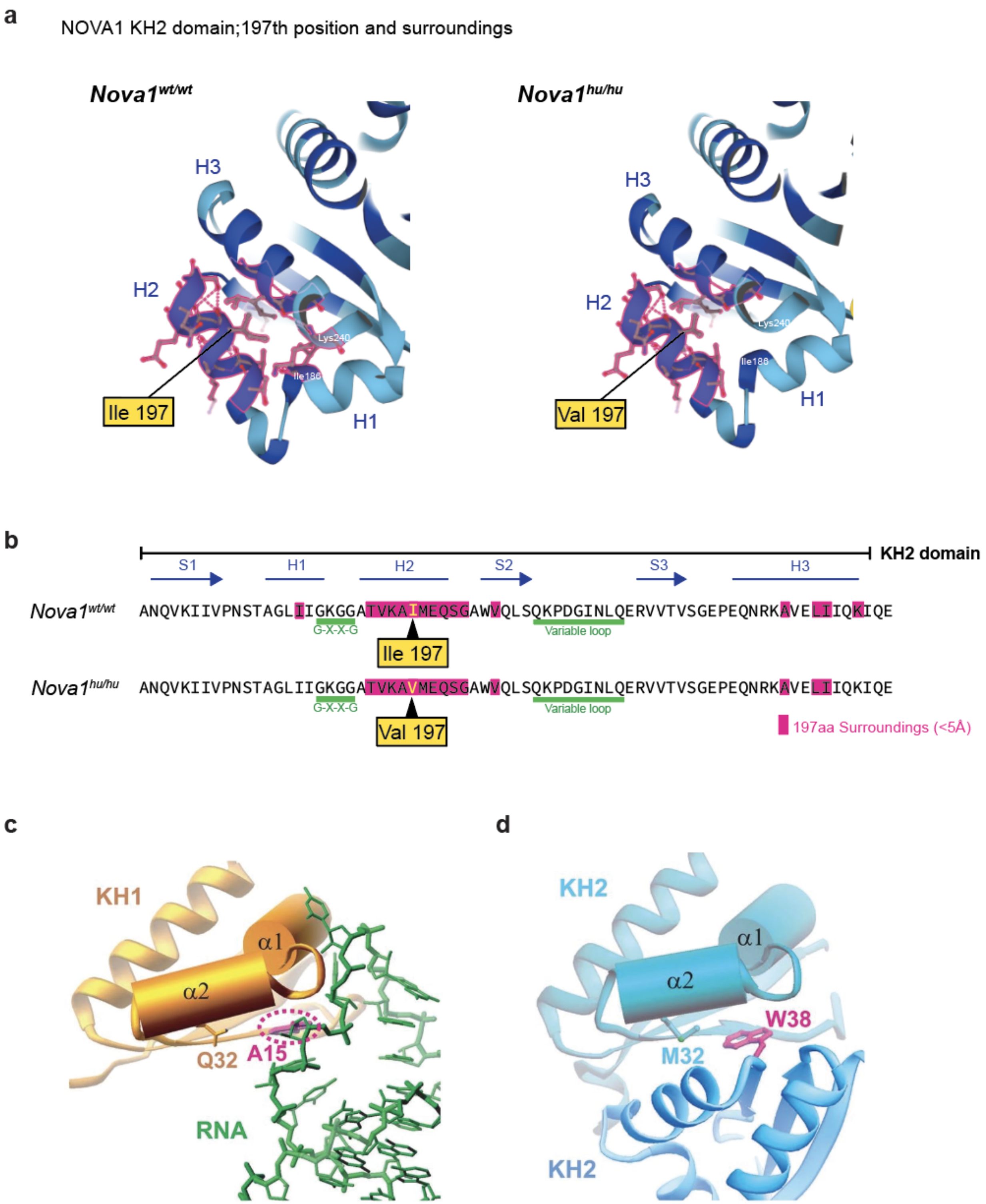

Supplementary Fig. 9

Predicted structural model caused by I197V substitution in the KH2 domain of NOVA1. (**a**) 3D structure prediction by AlphaFold2 (https://alphafold.ebi.ac.uk/), showing the expanded KH2 domain of NOVA1. The 197th amino acid is centered, and its proximal amino acids (<5 Å) are

colored in pink. In this model, the change from isoleucine to valine results in the loss of contact with the amino acid residues at H1 and H3 due to the loss of one carbon chain of the amino acid side chain. (**b**) Illustration of the amino acids surrounding I197V relative to the secondary structure sequence of KH2. Amino acids structurally proximal to the 197th amino acid (<5 Å) predicted by AlphaFold2 are colored in pink. Corresponding to Fig. 1e. (**c**-**d**) Structures of models based on crystallographic data, in which the 197th amino acid is adjacent to amino acids involved in KH-domain-RNA interactions (Q32 in **c**, equivalent to the relative position of amino acid 198 in KH1) or KH-domain-protein interactions (M32 in **d**, equivalent to amino acid 198 in KH2). The models are from Figure 9 of Teplova et al., 2011[5].

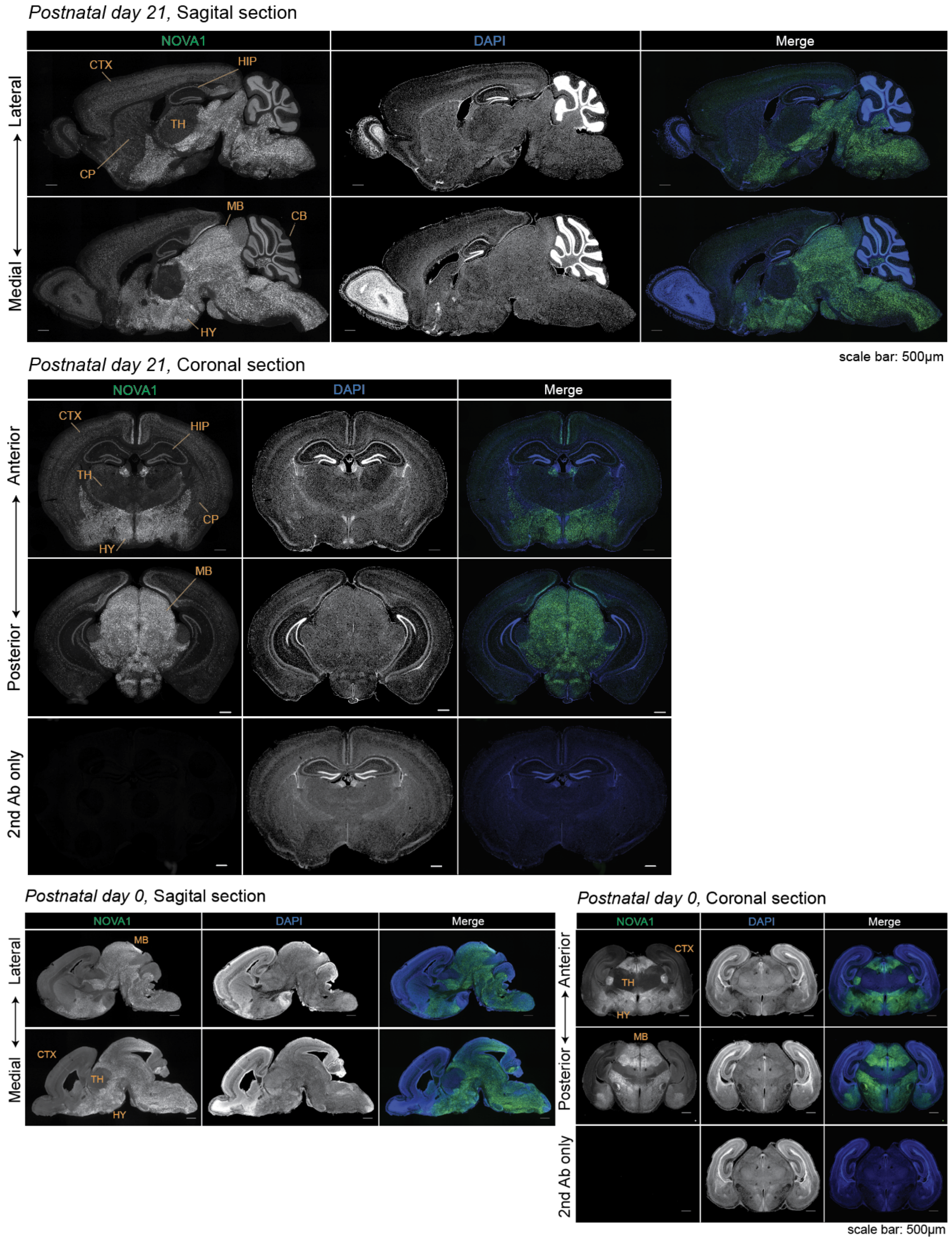

Postnatal day 21, Sagital section
NOVA1
DAPI
Merge
Lateral
Medial
CTX
HIP
TH
CP
MB
CB
HY
scale bar: 500μm
Postnatal day 21, Coronal section
NOVA1
DAPI
Merge
Anterior
Posterior
2nd Ab only
CTX
HIP
TH
CP
HY
MB
Postnatal day 0, Sagital section
NOVA1
DAPI
Merge
Lateral
Medial
MB
CTX
TH
HY
Postnatal day 0, Coronal section
NOVA1
DAPI
Merge
Anterior
Posterior
2nd Ab only
CTX
TH
HY
MB
scale bar: 500μm

Supplementary Fig. 10

NOVA1 immunostaining in the mouse brain. Immunostaining for NOVA1 (green) and DAPI (blue) in postnatal day 21 (P21) and 0 (P0) mouse brain. The scale bars indicate 500µm. The corresponding brain regions are indicated in the orange characters. CTX: cortex, HIP: hippocampus, CP: Caudate putamen, TH: thalamus, HY: hypothalamus, MB: midbrain, CB: cerebellum. The images of NOVA1 staining of P21 mouse brain are corresponding to Figure 3a.

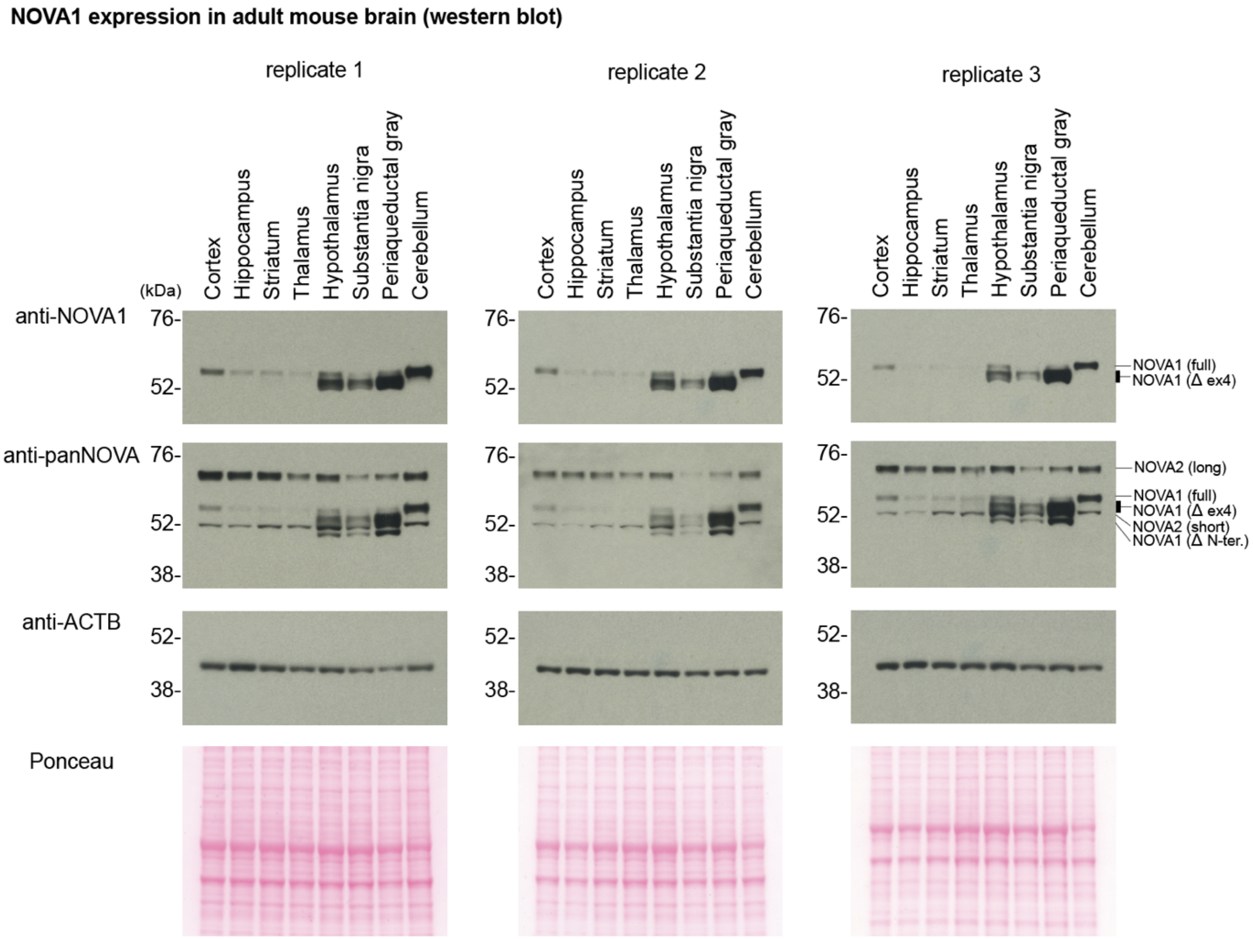

Supplementary Fig. 11

NOVA1 protein expression in dissected mouse brain. Western blotting for NOVA1, panNOVA and ACTB proteins in dissected brain regions of adult mouse brain. Proteins and isoforms corresponding to each band are listed on the right. The highly expressed NOVA1 in the hypothalamus, substantia nigra and periaqueductal gray is Exon4 minus isoform[6]. Three biological replicates are shown. The NOVA1 and ACTB plots in replicate 2 are corresponding to Fig 3b. Source data are provided as a Source Data file.

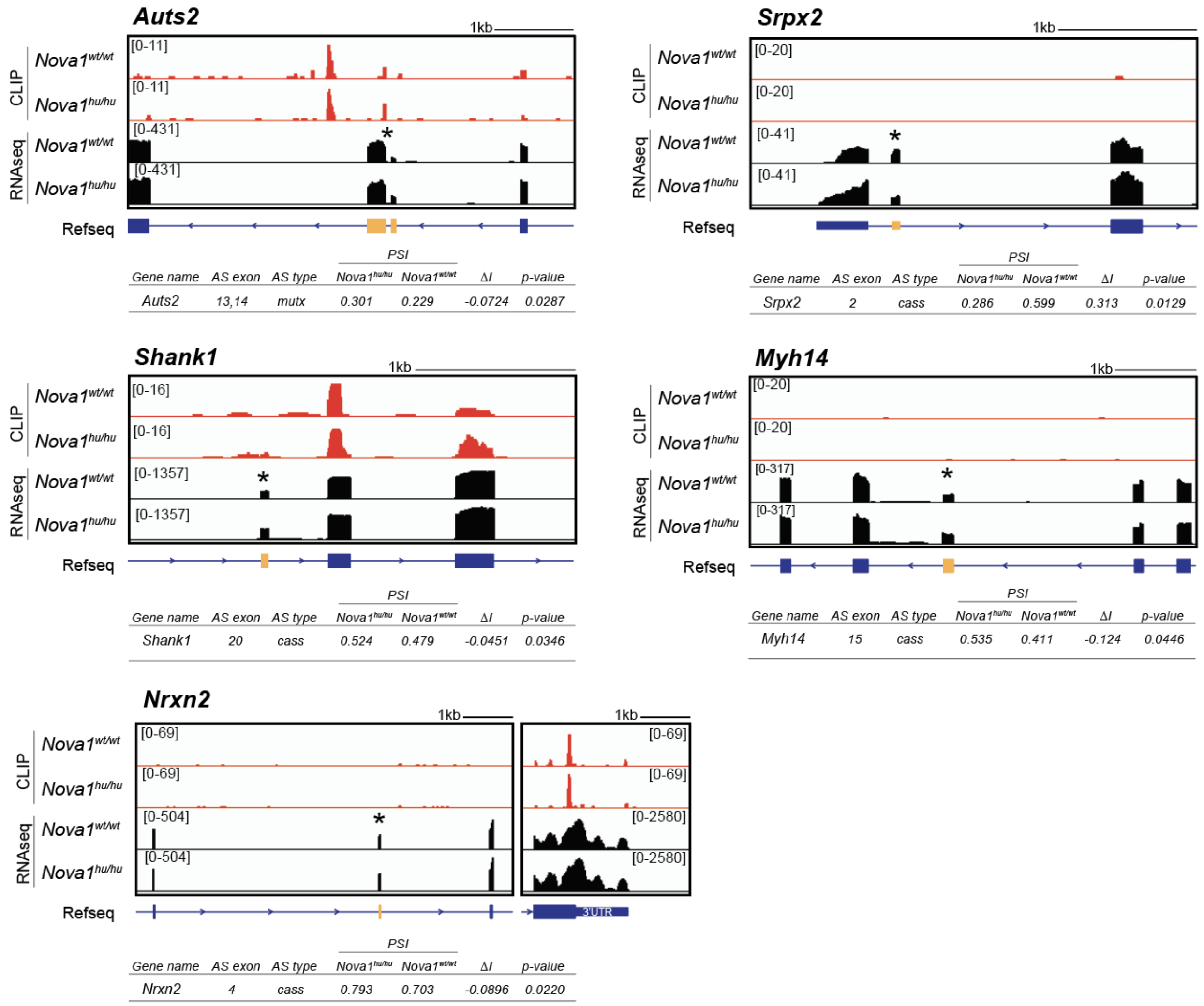

Supplementary Fig. 12

Vocal behavior related transcripts showing differential AS in *Nova1*$^{hu/hu}$ mice. AS exons are colored in yellow. Information of each AS event is described below the IGV snapshots; Gene name, AS exon number, AS type, percent spliced-in value (PSI, the percent of transcripts that include a specific AS exon), percent change (ΔI, ΔPSI; *Nova1*$^{hu/hu}$ vs. *Nova1*$^{wt/wt}$) and *p*-value. AS splicing events are classified into the following types: Cassette exon (cass), alternative 5’ splice site (alt5), alternative 3’ splice site (alt3), tandem cassette (taca), mutually exclusive exons (mutx).

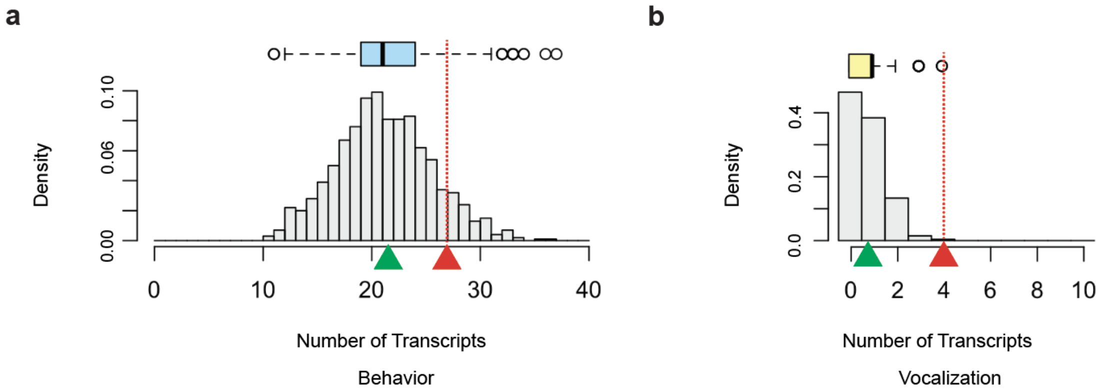

a

Number of Resamplings	Mean	Variance	SD	Median	First quartile	Third quartile
1000	21.6	19.3	4.4	21.0	19.0	24.0

Minimum	Maximum	Observed count	rank (%)
12.0	31.0	27.0	9.9 %

b

Number of Resamplings	Mean	Variance	SD	Median	First quartile	Third quartile
1000	0.7	0.6	0.8	1.0	0.0	1.0

Minimum	Maximum	Observed count	rank
0.0	2.0	4.0	< 4 %

Supplementary Fig. 13

The resampling analysis for differential AS events in *Nova1*$^{hu/hu}$ mice. (**a**) The 650 random resampling were repeated 1000 times from a list of transcripts detected in the RNAseq dataset to calculate the number of transcripts those annotated in the behavior category in gene ontology database (https://geneontology.org/). The histogram shows the density of the number of transcripts detected for each resampling, the boxplots at the top show the distribution features (median and quartiles (box) and maximum minimum (whiskers) and outliers (dots)). The green triangle indicates mean value (21.6) of the resampling. The red triangle indicates the number of transcripts detected in this study (27). The number of trials that exceeded the number of 27 was 99, 9.9% probability, indicating the number of transcripts detected in this study is higher than the average number of transcripts detected by chance. (**b**) The 27 random resampling were repeated 1000 times from the 843 transcripts annotated as the behavior category in the gene ontology database to calculate the number of transcripts those annotated in the vocalization category. The green triangle indicates the mean value (0.711) of the resampling. The red triangle indicates the number of transcripts detected in this study (4). Four trials detected the same number of transcripts as the observed number of transcripts 4, and zero trials exceeded 4, the probability is less than 4%, indicating the number of transcripts detected in this study is higher than the number of transcripts detected by chance with the 5% level of significance. Source data are provided as a Source Data file.

a

Vocalization behavior (GO: 0071625)

MGI Gene/Marker ID	Symbol	Name	Chr	P21.mid	P21.cx	P21.cb	e18.cx
MGI:1919847	Auts2	autism susceptibility candidate 2	5	✓	✓	✓	✓
MGI:1928478	Brinp1	bone morphogenic protein/retinoic acid inducible neural specific 1	4				✓
MGI:1923433	Celf6	CUGBP, Elav-like family member 6	9	✓			
MGI:1914047	Cntnap2	contactin associated protein-like 2	6	✓	✓	✓	✓
MGI:1277959	Dlg4	discs large MAGUK scaffold protein 4	11	✓	✓	✓	✓
MGI:894663	Ext1	exostosin glycosyltransferase 1	15	✓		✓	
MGI:1914004	Foxp1	forkhead box P1	6	✓	✓	✓	✓
MGI:2148705	Foxp2	forkhead box P2	6	✓	✓		
MGI:95729	Gli3	GLI-Kruppel family member GLI3	13				
MGI:3651981	Mup20	major urinary protein 20	4				
MGI:1919210	Myh14	myosin, heavy polypeptide 14	7				
MGI:107754	Neurog1	neurogenin 1	13			✓	
MGI:2444609	Nlgn3	neuroligin 3	X	✓			✓
MGI:3775191	Nlgn4l	neuroligin 4-like	XY				
MGI:1096391	Nrxn1	neurexin I	17	✓	✓	✓	✓
MGI:1096362	Nrxn2	neurexin II	19	✓	✓	✓	✓
MGI:1096389	Nrxn3	neurexin III	12	✓	✓	✓	✓
MGI:3613677	Shank1	SH3 and multiple ankyrin repeat domains 1	7	✓	✓	✓	✓
MGI:2671987	Shank2	SH3 and multiple ankyrin repeat domains 2	7	✓	✓	✓	✓
MGI:1930016	Shank3	SH3 and multiple ankyrin repeat domains 3	15	✓	✓	✓	✓
MGI:1916042	Srpx2	sushi-repeat-containing protein, X-linked 2	X				
MGI:2385852	Tifab	TRAF-interacting protein with forkhead-associated domain, family member B	13				

b

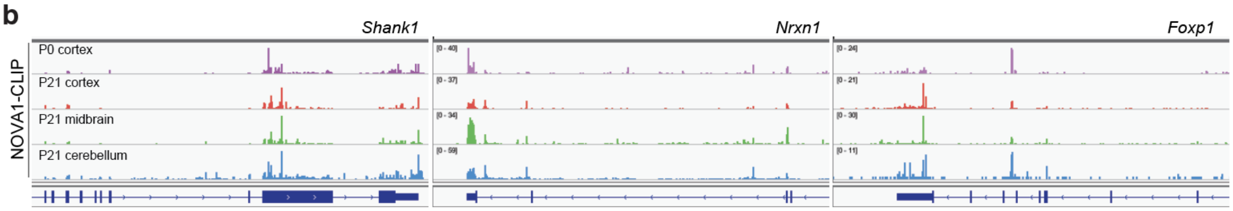

Supplementary Fig.14

NOVA1-CLIP binding peaks in vocalization related transcripts. (**a**) A list of genes classified to be involved in vocal behavior in gene ontology analysis. Genes for which NOVA1 binding was detected on the transcript in CLIP analysis were marked (check mark). The threshold for NOVA1 binding was a peak detected in all three biological replicates with peak height greater than 10. For P21 samples (cortex, midbrain, cerebellum), the gene was marked if it meets the above criteria in either *Nova1*$^{hu/hu}$ or *Nova1*$^{wt/wt}$. (**b**) Examples of vocalization-related genes with NOVA1 CLIP peaks on their transcripts.

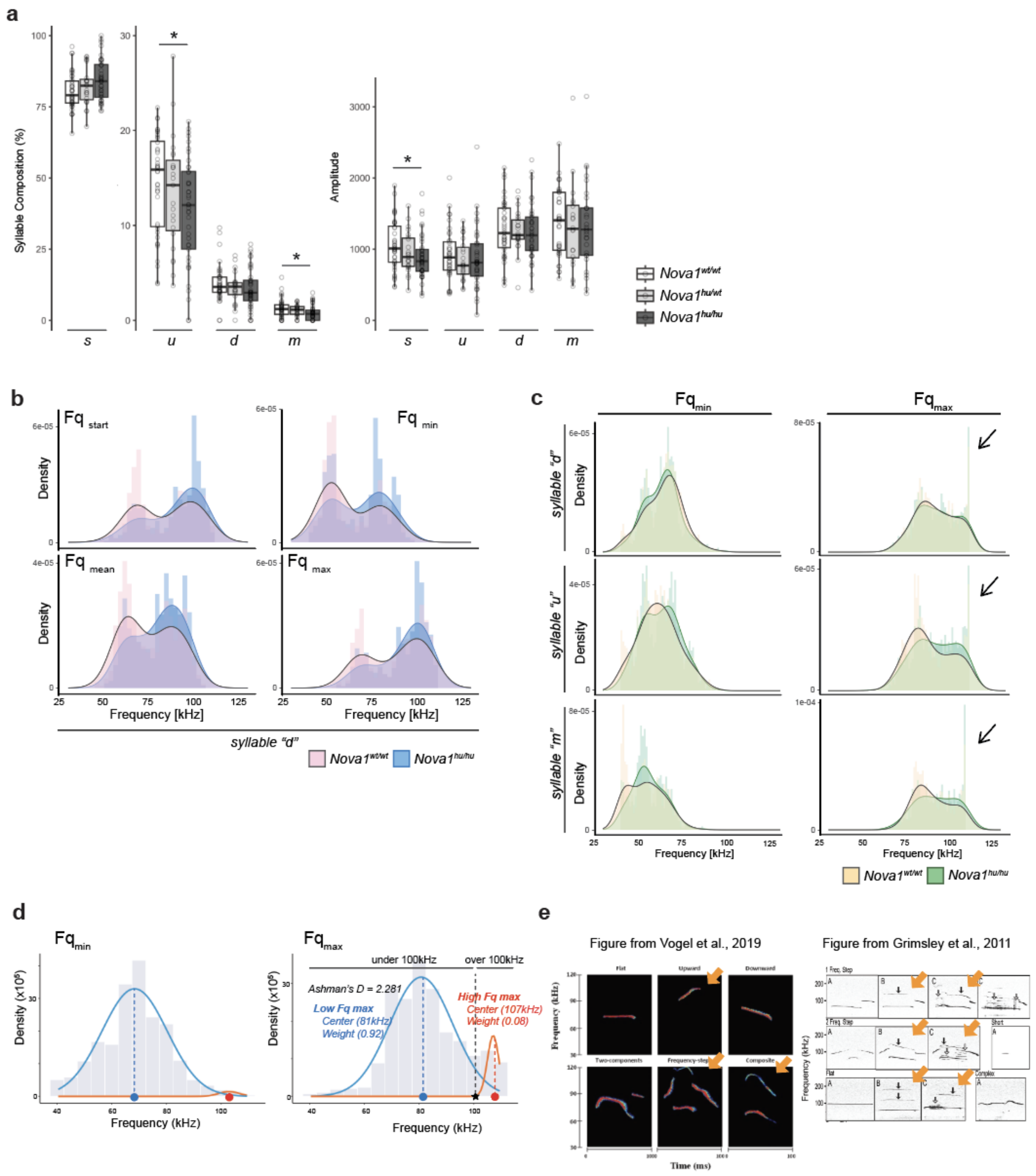

Supplementary Fig. 15

USV characteristics in pups and adults. (**a**) Syllable composition (left) and amplitude (right) for each syllable in pup USVs. Data is represented as boxplots. Each open circle indicates data from a single pup. *p*-values were calculated by Wilcoxon rank sum test and corrected with Bonferroni method. * *p* value <0.05. *Nova1*$^{hu/hu}$ N=41, *Nova1*$^{hu/wt}$ N=23, *Nova1*$^{wt/wt}$ N=40 pups. (**b**) Example of bimodal distributions in peak frequency (Fq) in pup USVs. Density plots of start, minimum,

mean, and maximum Fq for syllable "d" observed in each genotype. (**c**) Density plots of minimal and maximum Fq for jump syllables ("d", "u", "m") observed in each genotype. Arrows indicate high Fq_{max} syllables above 100kHz. (**d**) Density plots of Fq_{min} and Fq_{max} in adult USVs. Two Gaussians (blue and orange lines) fitted are overlaid. The Fq_{max} plot is corresponding to Figure 4h. The Gaussian centers (green and red circles) and weights are labeled. The black star indicates 100kHz cutoff of high and low Fq_{max} USVs. (**e**) Examples of mouse USVs spanning the high-frequency regions from previous studies. Figures from Vogel et.al., 2019[7]. and Grimsley et al., 2011[8]. USVs with signals above 100 kHz are indicated by orange arrows.

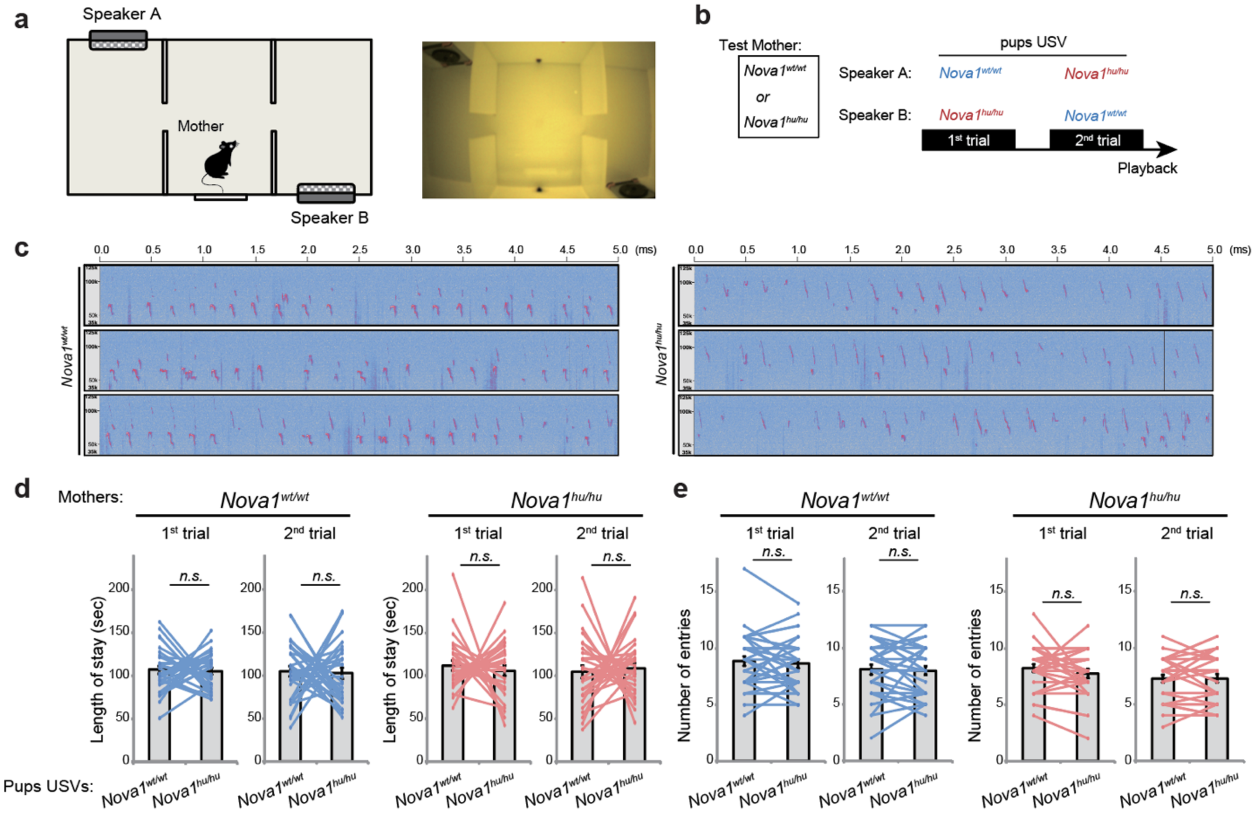

Supplementary Fig. 16

Playback behavioral experiments for pup USVs. (**a**) Apparatus used for neonatal mouse vocal selection tests with nursing mother mice. The box consists of three rooms connected by a passageway through which the mouse can pass, and a speaker is attached to each room at each end. (**b**) Overview of the neonatal mouse vocal selection test with mother mice. Nursing mother mouse of *Nova1*$^{wt/wt}$ or *Nova1*$^{hu/hu}$ was placed in the center of the room and their behavior was recorded while vocal recordings of neonatal mice were played. Vocal recordings of *Nova1*$^{wt/wt}$ or *Nova1*$^{hu/hu}$ neonatal mice were randomly played from speakers at both ends, respectively. To exclude direction preference, the recordings played from each speaker were switched after a one-minute break. (**c**) Vocal recordings of neonatal mice used in the experiments. The recordings were arranged from the data of the pup-USV test to reflect the overall parameters of each genotype (*Nova1*$^{wt/wt}$ or *Nova1*$^{hu/hu}$). (**d**) Comparison of the time the mother mouse stayed in the room where the recording of each neonatal mouse was played. Bars indicate mean ± standard error; dots indicate values for each individual. The time spent in each room by the same individual is connected by a line. (**e**) Comparison of the number of times the mother mouse entered the room where each neonatal mouse recording was played. Bars indicate mean ± standard error; dots indicate values for each individual. The time spent in each room by the same individual is connected by a line. *Nova1*$^{wt/wt}$ mother: N=32, *Nova1*$^{hu/hu}$ mother: N=29. Source data are provided as a Source Data file.

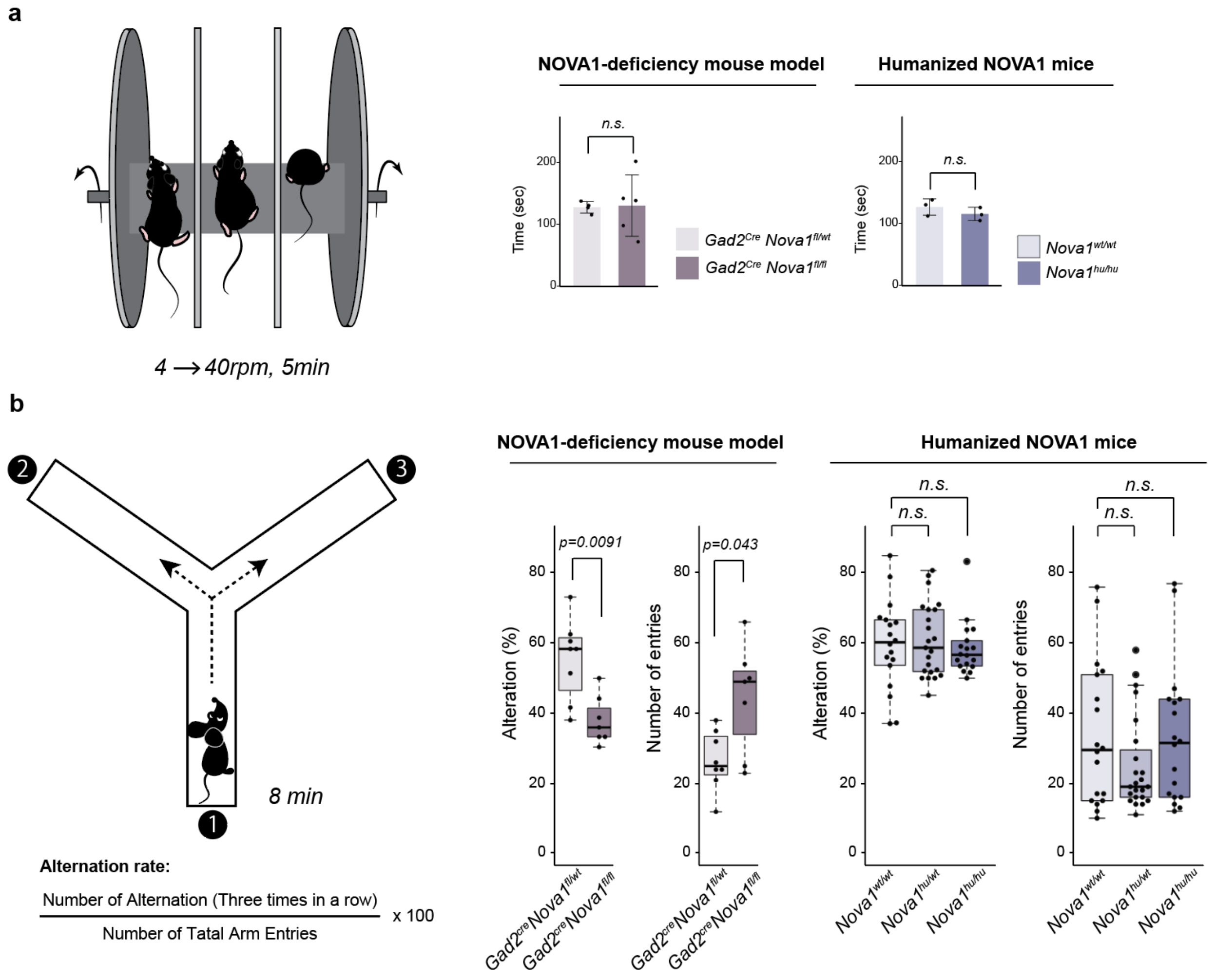

Supplementary Fig. 17

(**a**) Rotarod performance test. (left) illustration of the test. To measure the locomotor performance (motor coordination), mice were placed on an elevated revolving rod that accelerates at a constant rate (4 to 40rpm in 300sec). The time it took the animals to fall were recorded. Tests were performed three times and the average value were calculated. (middle) Results in NOVA1 deficiency mouse model (cKO mice: Tajima et al., 2023[9]). (right) Results in humanized NOVA1 mice. The bar graph represent mean ± standard deviation. The dot indicates average time for one mouse. $Gad2^{Cre}Nova1^{fl/wt}$ N=4, $Gad2^{Cre}Nova1^{fl/fl}$ N=5, $Nova1^{wt/wt}$ N=3, $Nova1^{hu/hu}$ N=3. Source data are provided as a Source Data file.

(**b**) Y-maze test. (left) illustration of the test and calculation for the alternation rate. Mice were allowed to freely explore a Y-shaped maze for 8 minutes. The number of entries into the arms and the number of triads were recorded to calculate the percentage of alternation. Alternations are consecutive entries into each arm of the Y-maze without any repeats (e.g., arm 1 -> 2 ->3). (middle) results in NOVA1 deficiency mouse model (cKO mice) The data is from Tajima et al., 2023[9]. (right) results in humanized NOVA1 mice. The alteration rates and total number of entries into arms during the tests are represented as boxplots with the minimum score, first quartile, median, third quartile, and maximum score. Each dot indicates data from a single mouse. $Gad2^{Cre}Nova1^{fl/wt}$

N=7, *Gad2*^{Cre}*Nova1*$^{fl/fl}$ N=8, *Nova1*$^{wt/wt}$ N=18, *Nova1*$^{hu/wt}$ N=23, Nova*1*$^{hu/hu}$ N=18. Source data are provided as a Source Data file.

Supplementary References

1. Schaeffer, S. W. Molecular population genetics of sequence length diversity in the Adh region of Drosophila pseudoobscura. *Genet. Res.* **80**, 163–175 (2002).
2. Meyer, M. *et al.* A High-Coverage Genome Sequence from an Archaic Denisovan Individual. *Science* **338**, 222–226 (2012).
3. Trujillo, C. A. *et al.* Reintroduction of the archaic variant of NOVA1 in cortical organoids alters neurodevelopment. *Science* **371**, (2021).
4. Lewis, H. A. *et al.* Sequence-specific RNA binding by a Nova KH domain: implications for paraneoplastic disease and the fragile X syndrome. *Cell* **100**, 323–332 (2000).
5. Teplova, M. *et al.* Protein-RNA and protein-protein recognition by dual KH1/2 domains of the neuronal splicing factor Nova-1. *Structure* **19**, 930–944 (2011).
6. Dredge, B. K., Stefani, G., Engelhard, C. C. & Darnell, R. B. Nova autoregulation reveals dual functions in neuronal splicing. *EMBO J.* **24**, 1608–1620 (2005).
7. Vogel, A. P., Tsanas, A. & Scattoni, M. L. Quantifying ultrasonic mouse vocalizations using acoustic analysis in a supervised statistical machine learning framework. *Sci. Rep.* **9**, 1–10 (2019).
8. Grimsley, J. M. S., Monaghan, J. J. M. & Wenstrup, J. J. Development of social vocalizations in mice. *PLoS One* **6**, e17460 (2011).
9. Tajima, Y. *et al.* NOVA1 acts on Impact to regulate hypothalamic function and translation in inhibitory neurons. *Cell Rep.* **42**, 112050 (2023).
10. Enard, W. *et al.* A Humanized Version of Foxp2 Affects Cortico-Basal Ganglia Circuits in Mice. *Cell* **137**, 961–971 (2009).
11. Hammerschmidt, K. *et al.* A humanized version of Foxp2 does not affect ultrasonic vocalization in adult mice. *Genes Brain Behav.* **14**, 583–590 (2015).
12. von Merten, S., Pfeifle, C., Künzel, S., Hoier, S. & Tautz, D. A humanized version of Foxp2 affects ultrasonic vocalization in adult female and male mice. *Genes Brain Behav.* **20**, e12764 (2021).

13. Saito, Y. *et al.* NOVA2-mediated RNA regulation is required for axonal pathfinding during development. *Elife* **5**, (2016).
