# Supplementary material for: A humanized NOVA1 splicing factor alters mouse vocal communications
Source: Nat Commun. 2025 Feb 18;16:1542. doi: 10.1038/s41467-025-56579-2 (PMC11836289; doi:10.1038/s41467-025-56579-2)
Supplement: Supplementary file 2 — Description of Aditional Supplementary File [file 41467_2025_56579_MOESM2_ESM.pdf]

Legends for Supplementary Data

Supplementary Data 1.

Tajima's D and normalized Tajima's D.

Tajima's D values and their normalized values were calculated based on the ExAC data sets. The genes on datasets 3 and 4 are based on the reports of Meyer et al[2], and the genes on dataset 5 are based on Trujillo et al[3]. See Methods for details on calculation of the Tajima's D values.

Supplementary Data 2.

Selection analysis for human-specific SNPs using CLUES2.

The columns display the log-likelihood ratio (logLR), the negative log10-transformed p-value (-log10(p-value)), and the selection coefficient (SelectionMLE1) for each SNP across a single epoch (0 to 200,000), calculated by CLUES2. The table is sorted by *p*-value, with SNPs highlighted in yellow, including NOVA1, showing stronger selection signals. Gene list and SNP information are from Trujillo et al., 2021[3].

Supplementary Data 3

Differential CLIP peaks between *Nova1*$^{wt/wt}$ and *Nova1*$^{hu/hu}$.

Peaks are extracted that fulfill the criteria of being detected in all three biological replicates and having tags per peak at least 10 (peak height>10) for either *Nova1*$^{hu/hu}$ or *Nova1*$^{wt/wt}$ mice for NOVA1-CLIP analysis in P21 midbrain. Peaks on lowly expressed transcripts in P21 midbrain are excluded using RNA sequencing data. NOVA1 binding peaks with *p*-value less than 0.01 and absolute value of $\log_2$FC more than 1 were shown. The table contains the number of read counts for each genotype along with the genomic location of each peak: gene name, chromosome, start, end, strand, and annotation on the transcript and peak ID. The statistical analysis was performed using edgeR. *Nova1*$^{wt/wt}$ N=3, *Nova1*$^{hu/hu}$ N=3.

Supplementary Data 4.

Differential alternative splicing (AS) events in *Nova1*$^{hu/hu}$ mice

List of events for which differences were detected in AS analysis in the midbrain of *Nova1*$^{hu/hu}$ and *Nova1*$^{wt/wt}$ mice (p-value<0.05, $|dI|$>0.05). For each event, the gene name, chromosome number,

genomic start and end position of the event, strand, splicing type, PSI values and its differences (ΔPSI, dI), *p*-value and ID information are listed. PSI: percent spliced-in value, the percent of transcripts that include a specific AS exon. ΔPSI: percent change in *Nova1*$^{hu/hu}$ vs. *Nova1*$^{wt/wt}$. AS splicing events are classified into the following types: Cassette exon (cass), alternative 5' splice site (alt5), alternative 3' splice site (alt3), tandem cassette (taca), mutually exclusive exons (mutx), intron retention (iret). The statistical analysis was performed using Quantas pipeline. *Nova1*$^{wt/wt}$ N=4, *Nova1*$^{hu/hu}$ N=4.

Supplementary Data 5.

Classification in transcripts belonging to behavioral gene ontology categories.

Detailed classification of 27 transcripts with differential AS events in the midbrain of P21 *Nova1*$^{hu/hu}$ mice. The 27 transcripts that were classified into behavioral categories in the gene ontology were divided according to the minor classification to which each transcript belongs. The major gene ontology term, ID, minor classification, and category are shown.

Supplementary Data 6.

Syllables detected in isolation induced USV test in pups.

The acoustic waveform data for each pup was processed by the Mouse Song Analyzer (from Erich Jarvis lab) to obtain values for each syllable: syllable type, duration (time per syllable [sec]), ISI (intersyllable interval), fqVariance (degree of variance), purity, amplitude (magnitude of loudness), bandwidth (width of peak frequency) and peak frequency (Fq) variabilities; fqmin (minimum), fqmean (mean), fqmax (maximum), fqstart (start), fqend (end). notIDd: not identified.

Supplementary Data 7.

USV features in each pup.

In isolation induced USV test for pup, the following USV features are calculated for each pup: number of USVs, call rate, percent.starting (percent of starting syllable type of sequence (continuous syllables)), percent.composition (percent of syllable), sequence length. For each syllable type ("s", "u", "d", "m"), following parameter are calculated: Bw (bandwidth), Amp (amplitude), fqVar (Fq variance), Purity, Dur (duration), and Fq variabilities; fqMin (minimum), fqMean (mean), fqMax (maximum), fqStart (start), fqEnd (end).

Supplementary Data 8.

USV features in each genotype of pup.

USV features in *Nova1*$^{hu/hu}$, *Nova1*$^{hu/wt}$ and *Nova1*$^{wt/wt}$ pups. Bw (bandwidth), Amp (amplitude), fqVar (Fq variance), Dur (duration). The values represent the mean value for each genotype. The standard error (se) values for each parameter are inserted in the adjacent mean columns. The parameters statistically different from control (*Nova1*$^{wt/wt}$) are indicated with asterisk (*$p < 0.05$). p-values were calculated by Wilcoxon rank sum test (two-sided, Bonferroni correction). *Nova1*$^{wt/wt}$ pup N=40, *Nova1*$^{hu/wt}$ pup N=23, *Nova1*$^{hu/hu}$ pup N=41.

Supplementary Data 9.

Bimodal distribution parameters at Fq_{max} (maximum frequency) in pup USVs

For each syllable type, the bimodality in Fq_{max} was assessed by Ashman's D test (Fig. 4c). Two Gaussians were fitted to calculate each distribution parameters: component (weight), Fq mean [Hz], Sd (standard deviation) and cutoff value (intersection point) [kHz]. The distributions are classified as Low Fq_{max} and High Fq_{max} by the cutoff.

Supplementary Data 10.

Proportion of high/low Fq_{max} in pup-USVs in each genotype.

The ratio of low Fq_{max} and high Fq_{max} in each syllable type are shown for each genotype. The values represent the mean value for each genotype. The standard deviation (sd) and the standard error (se) values for each parameter are inserted in the adjacent mean columns. The parameters statistically different from control (*Nova1*$^{wt/wt}$) are indicated with asterisk (*p<0.05, **p<0.01). p-values were calculated by Wilcoxon rank sum test (two-sided, Bonferroni correction). *Nova1*$^{wt/wt}$ pup N=40, *Nova1*$^{hu/wt}$ pup N=23, *Nova1*$^{hu/hu}$ pup N=41.

Supplementary Data 11.

Syllables detected in courtship induced USV test in adults.

The acoustic waveform data for each adult mouse was processed by the Mouse Song Analyzer (from Erich Jarvis lab) to obtain values for each syllable: syllable type, duration (time per syllable

[sec]), ISI (intersyllable interval), fqVariance (degree of variance), purity, amplitude (magnitude of loudness), bandwidth (width of peak frequency) and peak frequency (Fq) variabilities; fqmin (minimum), fqmean (mean), fqmax (maximum), fqstart (start), fqend (end). Day: recording day 1-3. The test was conducted three times per mouse, one week apart. LF: live female for stimulation. notIDd: not identified.

Supplementary Data 12.

USV features in each adult mouse.

In courtship induced USV test for adult mouse, the following USV features are calculated: number of USVs, call rate, percent.starting (percent of starting syllable type of sequence (continuous syllables)), percent.composition (percent of syllable), sequence length. For each syllable type ("s", "u", "d", "m"), following parameter are calculated: Bw (bandwidth), Amp (amplitude), fqVar (Fq variance), Purity, Dur (duration), and Fq variabilities; fqMin (minimum), fqMean (mean), fqMax (maximum), fqStart (start), fqEnd (end). The values were shown by each recording day per mouse (d1-d3).

Supplementary Data 13.

USV features in each genotype of adult mouse.

USV features in *Nova1*$^{hu/hu}$, *Nova1*$^{hu/wt}$ and *Nova1*$^{wt/wt}$ pups. Bw (bandwidth), Amp (amplitude), fqVar (Fq variance), Dur (duration, sec). The values represent the mean value for each genotype. The standard error (se) values for each parameter are inserted in the adjacent mean columns. The parameters statistically different from control (*Nova1*$^{wt/wt}$) are indicated with asterisk (*$p < 0.05$, ** $p < 0.01$). p-values were calculated by Wilcoxon rank sum test (two-sided, Bonferroni correction). *Nova1*$^{wt/wt}$ N=13, *Nova1*$^{hu/wt}$ N=14, *Nova1*$^{hu/hu}$ N=13.

Supplementary Data 14.

USV characteristics in long/ short duration "s" in adult-USVs in each genotype.

The "s" syllables were classified by the cutoff (44ms) into short or long duration (Fig. 4f). The values represent the mean value for each genotype. The standard error (se) values for each parameter are inserted in the adjacent mean columns. The parameters statistically different from control (*Nova1*$^{wt/wt}$) are indicated with asterisk (*p < 0.05, **p < 0.01). p-values were calculated by Wilcoxon rank sum test (two-sided, Bonferroni correction). *Nova1*$^{wt/wt}$ N=13, *Nova1*$^{hu/wt}$ N=14, *Nova1*$^{hu/hu}$ N=13.

Supplementary Data 15.

Proportion of syllable composition in high/low Fq_{max} in adult-USVs in each genotype.

The syllables were classified by the cutoff (100 kHz) into low or high Fq_{max} (Fig. 4h). The ratio of low Fq_{max} and high Fq_{max} in each syllable type are shown for each genotype. notIDd: not identified. The values represent the mean value for each genotype. The standard error (se) for each parameter is inserted in the adjacent mean columns. The parameters statistically different from control (*Nova1*$^{wt/wt}$) are indicated with asterisk (*p < 0.05). p-values were calculated by Wilcoxon rank sum test (two-sided, Bonferroni correction). *Nova1*$^{wt/wt}$ N=13, *Nova1*$^{hu/wt}$ N=14, *Nova1*$^{hu/hu}$ N=13.

Supplementary Data 16.

USV characteristics in low/ high Fq_{max} in adult-USVs in each genotype.

The syllables were classified by the cutoff (100kHz) into low or high Fq_{max} (Fig. 4h). The values represent the mean value for each genotype. The standard error (se) values for each parameter are inserted in the adjacent mean columns. The parameters statistically different from control (*Nova1*$^{wt/wt}$) are indicated with asterisk (*p < 0.05, **p < 0.01). p-values were calculated by Wilcoxon rank sum test (two-sided, Bonferroni correction). *Nova1*$^{wt/wt}$ N=13, *Nova1*$^{hu/wt}$ N=14, *Nova1*$^{hu/hu}$ N=13.

Supplementary Data 17.

Comparison of vocalization tests between humanized mouse models.

Comparison on USV tests between this study and three studies using humanized Foxp2 mice (Enard et al., 2009, Hammerschmidts et al., 2015, von Merten et al., 2021)[10–12]. The table contains experimental conditions (methods and analysis) and findings in each study. To avoid changing the nuance of the words in each report, the terms in each paper were quoted verbatim in the table (e.g., calls, elements, vocalizations).

Supplementary Data 18.

Expression changes in *Nova1$^{ko/ko}$* midbrain at E18.5.

Transcripts whose expression was affected in E18.5 midbrain in *Nova1* knockout mice were shown (*Nova1$^{wt/wt}$* vs. *Nova1$^{ko/ko}$*). RNA sequencing data are from Saito et al., 2016[13]. Corresponding values on the same transcripts in humanized NOVA1 mice at E18.5 midbrain are shown on the right column (*Nova1$^{wt/wt}$* vs. *Nova1$^{hu/hu}$*). Transcripts Per Kilobase Million (tpm) values represent the average value for each genotype. The statistical analysis was performed using edgeR. *Nova1$^{wt/wt}$* N=3, *Nova1$^{ko/ko}$* N=3 for Nova1 knockout mice comparison, *Nova1$^{wt/wt}$* N=6, *Nova1$^{hu/hu}$* N=6 for humanized Nova1 mice comparison.
