# Supplementary material for: A humanized NOVA1 splicing factor alters mouse vocal communications
Source: Nat Commun. 2025 Feb 18;16:1542. doi: 10.1038/s41467-025-56579-2 (PMC11836289; doi:10.1038/s41467-025-56579-2)
Supplement: Supplementary file 21 — Reporting Summary [file 41467_2025_56579_MOESM21_ESM.docx]

Yoko Tajima


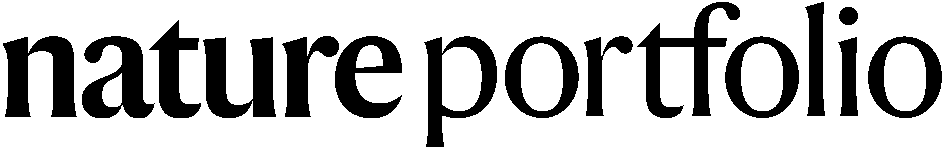
Corresponding author(s): Last updated by author(s):

Dec 11, 2024

Reporting Summary

Nature Portfolio wishes to improve the reproducibility of the work that we publish. This form provides structure for consistency and transparency in reporting. For further information on Nature Portfolio policies, see our Editorial Policies and the Editorial Policy Checklist.

Please do not complete any field with "not applicable" or n/a. Refer to the help text for what text to use if an item is not relevant to your study. For final submission: please carefully check your responses for accuracy; you will not be able to make changes later.

## Statistics

For all statistical analyses, confirm that the following items are present in the figure legend, table legend, main text, or Methods section.

n/a

x

x

Confirmed


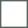
 The exact sample size (*n*) for each experimental group/condition, given as a discrete number and unit of measurement

x

x


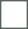
 A statement on whether measurements were taken from distinct samples or whether the same sample was measured repeatedly The statistical test(s) used AND whether they are one- or two-sided

*Only common tests should be described solely by name; describe more complex techniques in the Methods section.*


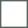
 A description of all covariates tested

x


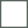
 A description of any assumptions or corrections, such as tests of normality and adjustment for multiple comparisons

A full description of the statistical parameters including central tendency (e.g. means) or other basic estimates (e.g. regression coefficient) AND variation (e.g. standard deviation) or associated estimates of uncertainty (e.g. confidence intervals)

x

For null hypothesis testing, the test statistic (e.g. *F*, *t*, *r*) with confidence intervals, effect sizes, degrees of freedom and *P* value noted

x


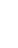

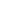

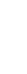

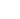

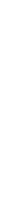

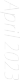


*Give P values as exact values whenever suitable.*

For Bayesian analysis, information on the choice of priors and Markov chain Monte Carlo settings

x

x

For hierarchical and complex designs, identification of the appropriate level for tests and full reporting of outcomes Estimates of effect sizes (e.g. Cohen's *d*, Pearson's *r*), indicating how they were calculated

x

*Our web collection on statistics for biologists contains articles on many of the points above.*

## Software and code

Policy information about availability of computer code Data collection

ExAC dataset (v0.3.1), refsnnp dataset (v b156), the Draft Neanderthal Genome Project (<https://www.ebi.ac.uk/ena/browser/view/PRJEB2065>), Denisovan Genome Project (http://cdna.eva.mpg.de/denisova/), cryostat (CM3050S, LEICA), microscope (BZ-X700, KEYENCE), film processor (SRX-101A, Konica), NovaSeq 6000 (illumina), MiSeq (illumina). Vocalizations were recorded with UltraSoundGateCM16/CMPA ultrasonic microphones connected to an Ultrasound Gate USGH amplifier. Recordings were saved using the AvisoftRecorderUSG software. All acoustic hardware was obtained from Avisoft Bioacoustics®.

The evolutionary statistic was performed with custom code. All script and steps are found on a GitHub repository (<https://github.com/cafeblue/popgen_dbsnp>). Acoustic waveforms were processed using a custom Python program called “Mouse Song Analyzer 2” (MSA2) ([https://github.com/Neurogenetics-Jarvis/MSA2](http://jarvislab.net/research/mouse-vocal-communication/)(Stoumpou)). Version of tools used in the analysis: bcftools (v1.19), OLego (v1.1.7), Quantas (v1.0.9), R (v4.2.0), Integrative Genomic Viewer (IGV, v2.13.0), Metascape (v3.5.20240101), CLIP Tool Kit (CTK, v1.1.3), novoalign (v3.09.02), HOMER (v4.11), ImageJ (v1.53), Burrows-Wheeler Aligner (BWA, v0.7.17-r1188), Picard (v2.18.7), Genome Analysis Toolkit (GATK, v4.4.0.0), ANNOVAR (v2).

Data analysis

For manuscripts utilizing custom algorithms or software that are central to the research but not yet described in published literature, software must be made available to editors and reviewers. We strongly encourage code deposition in a community repository (e.g. GitHub). See the Nature Portfolio guidelines for submitting code & software for further information.

## Data

Policy information about availability of data

All manuscripts must include a data availability statement. This statement should provide the following information, where applicable:

- Accession codes, unique identifiers, or web links for publicly available datasets
- A description of any restrictions on data availability
- For clinical datasets or third party data, please ensure that the statement adheres to our policy

Source data are provided with this paper. The sequencing data generated in this study have been deposited in the GEO database under the SuperSeries GSE253298 comprising Subseries GSE253296 and GSE253297 [https://www.ncbi.nlm.nih.gov/geo/query/acc.cgi?acc=GSE253298]. The vocalization data generated in this study are provided in the Supplementary Data file. The sequencing data for *Nova1* knockout mouse used in this study are available in the GEO database under accession code GSE69711 [https://www.ncbi.nlm.nih.gov/geo/query/acc.cgi?acc=GSE69711].


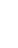

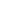

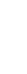

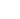

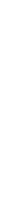

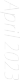


## Research involving human participants, their data, or biological material

Policy information about studies with human participants or human data. See also policy information about sex, gender (identity/presentation), and sexual orientation and race, ethnicity and racism.

Reporting on sex and gender

Reporting on race, ethnicity, or other socially relevant groupings

Population characteristics

Recruitment

Ethics oversight

Note that full information on the approval of the study protocol must also be provided in the manuscript.

# Field-specific reporting

Please select the one below that is the best fit for your research. If you are not sure, read the appropriate sections before making your selection.


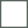
 Life sciences
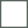
 Behavioural & social sciences
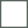
 Ecological, evolutionary & environmental sciences

x

For a reference copy of the document with all sections, see nature.com/documents/nr-reporting-summary-flat.pdf

# Life sciences study design

All studies must disclose on these points even when the disclosure is negative. Sample size

No statistical tests were used to predetermine sample size. Sample sizes were determined based on previously published work and literature in the field that have conducted similar experiments. For molecular experiments, sample sizes of 3-6 (biological replicates) were used. For in vivo experiments sample sizes of greater than 8 were used to account for the increased variability. Exact numbers were pre-determined based on the experimental design, number of animals available, and the animal housing conditions.

No data were excluded from the analyses.

Data exclusions

Replication

A minimum of three biological replicates are used in all experiments. The western blot and immunostaining are replicated for at least 3 times, and data are representative of these independent experiments.

Mice were assigned to each group based on genotype. Mice were age and sex matched for all experiments.

Randomization

Blinding

For the molecular experiments, the investigators were not blinded because all measurement modalities were quantitative. For the behavioral experiments, the investigators were blinded during the data collection. All samples and experiments were performed in the same way regardless of group or treatment.

# Behavioural & social sciences study design

All studies must disclose on these points even when the disclosure is negative.

Study description

Research sample

Sampling strategy

Data collection

Timing

Data exclusions

Non-participation

Randomization


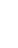

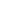

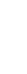

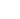

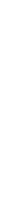

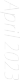


# Ecological, evolutionary & environmental sciences study design

All studies must disclose on these points even when the disclosure is negative.

Study description

Research sample

Sampling strategy

Data collection

Timing and spatial scale

Data exclusions

Reproducibility

Randomization

Blinding

Did the study involve field work? Yes No

## Field work, collection and transport

Field conditions

Location

Access & import/export

Disturbance

# Reporting for specific materials, systems and methods

We require information from authors about some types of materials, experimental systems and methods used in many studies. Here, indicate whether each material, system or method listed is relevant to your study. If you are not sure if a list item applies to your research, read the appropriate section before selecting a response.

Materials & experimental systems Methods

x

x

x

x

x

x

x

x

x

x

n/a Involved in the study Antibodies Eukaryotic cell lines

Palaeontology and archaeology Animals and other organisms Clinical data

Dual use research of concern

Plants

n/a Involved in the study

ChIP-seq

Flow cytometry

MRI-based neuroimaging

## Antibodies

Antibodies used

Primary antibodies used for immunohistochemistry and western blotting were as follows; rabbit anti-NOVA1 (1/1000 dilution) [EPR13847] (ab183024, abcam), rabbit anti-NOVA1 C-terminal (1/1000 dilution) [EPR13848] (ab183723, abcam), human anti-pan NOVA (1/10,000 dilution) (anti-Nova paraneoplastic human serum) and rabbit anti-ATCB (1/10,000 dilution) (ab8227, abcam).

Validation

rabbit anti-NOVA1 [EPR13847] (ab183024, abcam): <https://www.abcam.com/en-us/products/primary-antibodies/nova1-antibody-epr13847-ab183024?srsltid=AfmBOorV1lpoU96icGnevjhiw-muboB_kAcDeOpTS05rjWeC0AQ1y8J->

rabbit anti-NOVA1 C-terminal [EPR13848] (ab183723, abcam): <https://www.abcam.com/en-us/products/primary-antibodies/nova1-antibody-epr13848-c-terminal-ab183723?srsltid=AfmBOorL_-1q73eRu24DeXZHuebnhdqX1CqRPTf-1lj1yLfTQGPEvIqJ>

human anti-pan NOVA (anti-Nova paraneoplastic human serum) : Buckanovich, R. J., Posner, J. B. & Darnell, R. B. Nova, the paraneoplastic Ri antigen, is homologous to an RNA-binding protein and is specifically expressed in the developing motor system. Neuron 11, 657–672 (1993).

rabbit anti-ATCB (ab8227, abcam): <https://www.abcam.com/en-us/products/primary-antibodies/beta-actin-antibody-ab8227?srsltid=AfmBOoogyvWuoTKu5l6mae2XfTE3jqEUx4nUlMIlLY9yp9fKpCA0-KmQ>


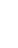

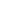

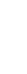

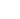

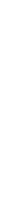

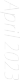


## Eukaryotic cell lines

Policy information about cell lines and Sex and Gender in Research Cell line source(s)

Authentication

Mycoplasma contamination Commonly misidentified lines

(See ICLAC register)

## Palaeontology and Archaeology

Specimen provenance

Specimen deposition

Dating methods

Tick this box to confirm that the raw and calibrated dates are available in the paper or in Supplementary Information.

Ethics oversight

Note that full information on the approval of the study protocol must also be provided in the manuscript.

## Animals and other research organisms

Policy information about studies involving animals; ARRIVE guidelines recommended for reporting animal research, and Sex and Gender in Research

Male and female wild type mice C57BL/6J (stock no. 000664) mice. Male and female Nova1hu/hu mice and their littermate controls (Nova1hu/wt and Nova1wt/wt mice) on C57BL/6J background. Mice were housed in a 12-h light/dark cycle, up to 5 mice per cage. Male or female mice aged 7 days (for isolation induced pup USV test) and 8-20 weeks (for playback behavioral experiment and courtship induced adult USV test) were used for animal experiments, as described. Littermates of the same-sex were randomly assigned to experimental groups.

Laboratory animals

Wild animals

No wild animals were used in the study.

Reporting on sex

The transcriptome data (RNA sequencing and AS analysis) used in this study included equal numbers of male-female pairs. For CLIP, immunostaining and Western blot analyses, same-sex genotype pairs were used for comparison. For vocalization tests, data from 7-day-old pups were used for analysis without distinction between males and females. For adult courtship-induced vocalization tests, due to the nature of the experimental method (males are the primary source of vocalizations), this was specified as an analysis of male vocalization characteristics.

n/a

Field-collected samples

Ethics oversight

All procedures were performed according to the guidelines of the Institutional Animal Care and Use Committee (IACUC) under the IACUC protocol # 23014 at the Rockefeller University. Reported in Methods section, under Animal experiments subsection.

Note that full information on the approval of the study protocol must also be provided in the manuscript.

## Clinical data

Policy information about clinical studies

All manuscripts should comply with the ICMJE guidelines for publication of clinical research and a completed CONSORT checklist must be included with all submissions.

Clinical trial registration

Study protocol

Data collection

Outcomes

## Dual use research of concern

Policy information about dual use research of concern

### Hazards

Could the accidental, deliberate or reckless misuse of agents or technologies generated in the work, or the application of information presented in the manuscript, pose a threat to:


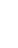

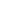

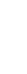

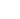

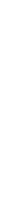

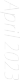


Public health

No Yes


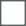
 National security


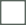
 Crops and/or livestock
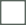
 Ecosystems


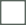
 Any other significant area

### Experiments of concern

Does the work involve any of these experiments of concern:

Demonstrate how to render a vaccine ineffective

No Yes

Confer resistance to therapeutically useful antibiotics or antiviral agents Enhance the virulence of a pathogen or render a nonpathogen virulent Increase transmissibility of a pathogen

Alter the host range of a pathogen

Enable evasion of diagnostic/detection modalities Enable the weaponization of a biological agent or toxin

Any other potentially harmful combination of experiments and agents

## Plants

Seed stocks

Novel plant genotypes

Authentication

## ChIP-seq

### Data deposition


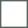
 Confirm that both raw and final processed data have been deposited in a public database such as GEO. Confirm that you have deposited or provided access to graph files (e.g. BED files) for the called peaks.

Data access links

*May remain private before publication.*

Files in database submission Genome browser session

(e.g. UCSC)

### Methodology

Replicates

Sequencing depth

Antibodies

Peak calling parameters

Data quality


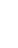

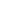

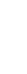

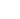

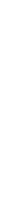

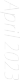


Software

## Flow Cytometry

### Plots

Confirm that:


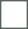
 The axis labels state the marker and fluorochrome used (e.g. CD4-FITC).


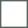
 The axis scales are clearly visible. Include numbers along axes only for bottom left plot of group (a 'group' is an analysis of identical markers).
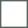
 All plots are contour plots with outliers or pseudocolor plots.


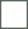
 A numerical value for number of cells or percentage (with statistics) is provided.

### Methodology

Sample preparation

Instrument

Software

Cell population abundance

Gating strategy

Tick this box to confirm that a figure exemplifying the gating strategy is provided in the Supplementary Information.

## Magnetic resonance imaging

### Experimental design

Design type

Design specifications

Behavioral performance measures

Imaging type(s)

Field strength

Sequence & imaging parameters

Area of acquisition

Diffusion MRI
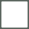
 Used
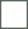
 Not used

### Preprocessing

Preprocessing software

Normalization

Normalization template

Noise and artifact removal

Volume censoring

### Statistical modeling & inference

Model type and settings

Effect(s) tested

Specify type of analysis:
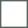
 Whole brain
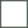
 ROI-based
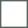
 Both Statistic type for inference

(See Eklund et al. 2016)

Correction

### Models & analysis

n/a

Involved in the study


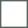
 Functional and/or effective connectivity
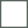
 Graph analysis

Multivariate modeling or predictive analysis

Functional and/or effective connectivity


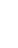

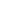

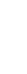

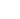

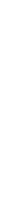

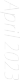


Graph analysis

Multivariate modeling and predictive analysis


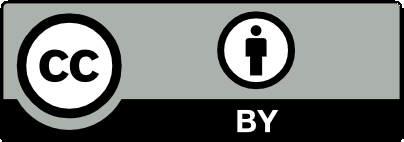
This checklist template is licensed under a Creative Commons Attribution 4.0 International License, which permits use, sharing, adaptation, distribution and reproduction in any medium or format, as long as you give appropriate credit to the original author(s) and the source, provide a link to the Creative Commons license, and indicate if changes were made. The images or other third party material in this article are included in the article's Creative Commons license, unless indicated otherwise in a credit line to the material. If material is not included in the article's Creative Commons license and your intended use is not permitted by statutory regulation or exceeds the permitted use, you will need to obtain permission directly from the copyright holder. To view a copy of this license, visit <http://creativecommons.org/licenses/by/4.0/>
